# Modulation Effect on Tubulin Polymerization, Cytotoxicity and Antioxidant Activity of 1H-Benzimidazole-2-Yl Hydrazones

**DOI:** 10.3390/molecules28010291

**Published:** 2022-12-29

**Authors:** Maria Argirova, Maya Guncheva, Georgi Momekov, Emiliya Cherneva, Rositsa Mihaylova, Miroslav Rangelov, Nadezhda Todorova, Petko Denev, Kameliya Anichina, Anelia Mavrova, Denitsa Yancheva

**Affiliations:** 1 Institute of Organic Chemistry with Centre of Phytochemistry, Bulgarian Academy of Sciences, 1113 Sofia, Bulgaria; 2 Faculty of Pharmacy, Medical University of Sofia, 1000 Sofia, Bulgaria; 3 Institute of Biodiversity and Ecosystem Research, Bulgarian Academy of Sciences, 1113 Sofia, Bulgaria; 4 Department of Organic Synthesis, University of Chemical Technology and Metallurgy, 1756 Sofia, Bulgaria

**Keywords:** benzimidazole, anticancer, antioxidant, tubulin polymerization, molecular docking, drug-likeness

## Abstract

1H-benzimidazol-2-yl hydrazones with varying hydroxy and methoxy phenyl moieties were designed. Their effect on tubulin polymerization was evaluated in vitro on porcine tubulin. The compounds elongated the nucleation phase and slowed down the tubulin polymerization comparably to nocodazole. The possible binding modes of the hydrazones with tubulin were explored by molecular docking at the colchicine binding site. The anticancer activity was evaluated against human malignant cell lines MCF-7 and AR-230, as well as against normal fibroblast cells 3T3 and CCL-1. The compounds demonstrated a marked antineoplastic activity in low micromolar concentrations in both screened in vitro tumor models. The most active were the trimethoxy substituted derivative **1i** and the positional isomers **1j** and **1k**, containing hydroxy and methoxy substituents: they showed IC_50_ similar to the reference podophyllotoxin in both tumor cell lines, accompanied with high selectivity towards the malignantly transformed cells. The compounds exerted moderate to high ability to scavenge peroxyl radicals and certain derivatives—**1l** containing *metha*-hydroxy and *para*-methoxy group, and **1b-e** with di/trihydroxy phenyl moiety, revealed HORAC values high or comparable to those of well-known phenolic antioxidants. Thus the 1H-benisimidazol-2-yl hydrazones with hydroxy/methoxy phenyl fragments were recognized as new agents exhibiting promising combined antioxidant and antineoplastic action.

## 1. Introduction

**Figure 1:**
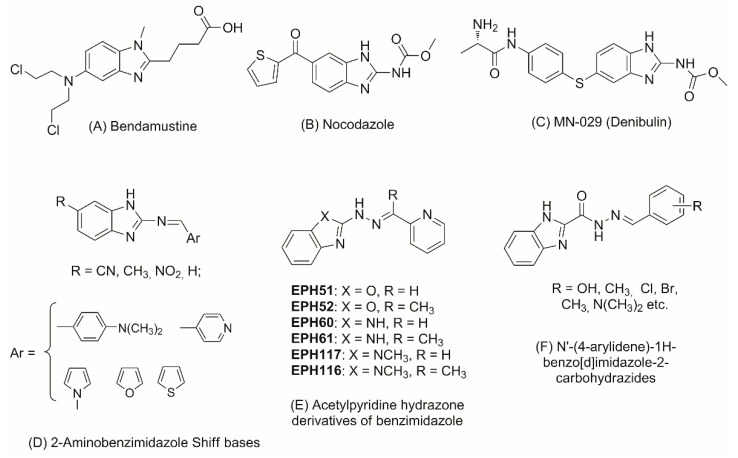
Benzimidazole derivatives with anticancer activity.

The potential of the benzimidazole architecture for development of novel antineoplastic compounds is well known. Many benzimidazoles have found application in the therapeutic practice—bendamustine, nocodazole, denibulin, etc. (Figure 1A–C). 2-Aminobenzimidazole heterocycle is a structural fragment frequently encountered in these drugs and it is of main importance as a precursor for the synthesis of new benzimidazoles with antitumor activity [1,2]. The structural modification of 2-amino group into azomethyne fragment has yielded a series of Schiff bases of 2-aminobenzimidazole (Figure 1D). They have shown cytotoxic activity toward several cancer cell lines: SW707 (colorectal carcinoma/rectal adenocarcinoma), HCV29T (bladder cancer), A549 (A549 (non-small cell lung carcinoma), T47D (breast cancer) and PC-3 [3,4]. 2-Acetylpyridine hydrazones of benzimidazole (Figure 1E) are another group of structural derivatives which exhibited potent cytotoxic activity against the growth of suspended leukemia and lymphomas and a number of solid tumor cell lines—HeLa, SOS (bone osteosarcoma), MCF-7 (breast cancer), MB9812 and A549 (lung cancer) [5]. Among them, 1-methylbenzimidazol-2-yl hydrazone (EPH116) was outlined as leading structure due to its potent inhibition of colon carcinoma and melanoma cells [5]. In view of this prominent antitumor activity, novel analogues of EPH116 were further synthesized by introducing electron donating or electron withdrawing groups at different positions of the 2-acetylpyridinium moiety [6].

Incorporation of acylhydrazone fragments at C2 position of the benzimidazole ring also has led to new compounds with anticancer activity as evidenced by the N′-(4-arylidene)-1H-benzo[d]imidazole-2-carbohydrazides (Figure 1F). The compounds showed cytotoxic effect against murine leukemia (L1210), human T-cell leukemia (CEM), human cervix carcinoma (HeLa), human pancreas carcinoma cells (Mia Paca-2) in the low micromolar range [7]. 

Oxidative stress leads to cellular damage and indirectly might induce DNA mutations, gene instability and cellular proliferation thus contributing to carcinogenesis [8,9,10,11]. It is known that ROS act as a secondary precursor in the intracellular signaling pathway that induce and maintain the oncogenic phenotype of the cancer cells. At the same time ROS are able to induce aging and apoptosis which contributes to their anti-tumorogenic effect. The increased generation of ROS is characteristic for cancer cells where changes in the signal transduction are observed. The DNA mutations have a key role in carcinogenesis, evidence of which are the increased levels of oxidative DNA changes (formation of 8-hydroxyguanine, 8-OH-G). In this regard, the antioxidants might contribute as a useful therapeutic option in the fight against cancer. The advantages of therapy that combines antineoplastic with antioxidant action are highlighted by the broad studies of melatonin [12]. Due to its antioxidant effect and free radical scavenging properties melatonin can provide protection against damage from carcinogenic substances [13,14,15,16]. On the other hand, it exerts also a direct anticancer action by inhibiting the proliferation and growth of tumor cells, inducing cellular turnover and replacement of tumor cells with healthy cells through apoptosis [12,17,18,19,20,21,22,23]. Having in mind the scientific data related to the connection between the oxidative stress and the processes of carcinogenesis, it is of interest to study compounds with antioxidant properties as potential antineoplastic agents.

**Figure 2:**
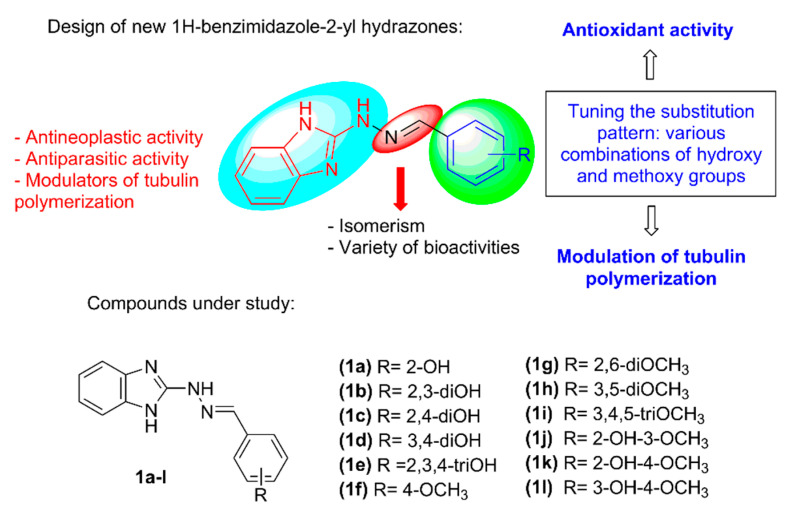
Molecular structure design of 1H-benzimidazole-2-yl hydrazones and compounds under study.

Recently a series of 1H-benzimidazole-2-yl hydrazones (Figure 2), containing fluoro-, hydroxy- and methoxy-substituted phenyl rings or 1,3-benzodioxolyl moiety were synthesized and demonstrated potent larvicidal effect against isolated muscle larvae of *T. spiralis* as well as moderate antiproliferative activity against MCF-7 breast cancer cells [24,25]. It was also shown that the compounds are able to inhibit tubulin polymerization in vitro [24]. Further studies outlined that the introduction of hydroxyphenyl and methoxyphenyl moieties in the 1H-benzimidazol-2-yl hydrazone structure (**1a-l**, Figure 2) is beneficial for the development of new anthelmintic agents showing radical scavenging properties in addition [25]. The hydroxy/methoxy derivatives exhibited a significant in vitro effect against stable free radicals—DPPH and ABTS, as well as iron induced oxidative damage in model systems containing lecithin and deoxyribose. By means of computational methods, it was shown that the 1H-benzimidazole-2-yl hydrazones possess very versatile radical scavenging properties—several reaction sites and possibility to act simultaneously through several possible reaction pathways with variety of free radicals: ^●^OCH_3_, ^●^OOH and ^●^OOCH_3_.

These promising results motivated us to broaden the study of the synthesized hydroxy/methoxy substituted 1H-benzimidazol-2-yl hydrazones **1a–l** (Figure 2) by investigating their cytotoxic effect towards MCF-7 (ER-positive breast adenocarcinoma) and AR-230 (chronic myeloid leukemia) cell lines, ability to interfere with tubulin polymerization as possible pharmacological target and some further aspects of their antioxidant action. The selectivity of the compounds towards the cancerous cells was evaluated by screening the toxicity towards the normal cell line 3T3 (mouse fibroblasts) and CCL-1 (murine fibroblasts). The possible modes of interaction with tubulin were explored by molecular docking in the colchicine binding site of the tubulin dimer. Physico-chemical properties and drug-likeness of the compounds were estimated based on several structural parameters.

## 2. Results and Discussion

### 2.1. Synthesis

The 1H-benzimidazole-2-yl hydrazones can be conveniently synthesized in a four-step reaction pathway [24,25]. Following this reaction pathway, benzimidazole-2-thione was obtained from o-phenylenediamine, potassium hydroxide and carbon disulfide and then oxidized to the corresponding 1H-benzimidazol-2-yl-sulfonic acid with alkaline solution of KMnO_4_. Nucleophilic substitution of the sulfo group with hydrazine hydrate afforded the 1H-benzimidazol-2-yl hydrazine which was coupled with various hydroxy or/and methoxy substituted benzaldehydes to the target hydrazone derivatives **1a–l** (Figure 2). The synthesis and product identification are described in more detail in a previous publication [25].

### 2.2. In Vitro Effect on Tubulin Polymerization and Docking Study on the Tubulin-Ligands Interactions 

The ability of the compounds to modulate the polymerization of tubulin was evaluated in vitro on purified porcine tubulin by spectrophotometric monitoring at 340 nm and compared to those of nocodazole and paclitaxel as reference compounds. Nocodazole which inhibits the assembly of tubulin was used as a positive control, and paclitaxel which is a stabilizing agent of tubulin polymerization was used as a negative control. Tubulin polymerization was determined with 10 µM of the reference drugs and compounds 1**a–l** for 90 min.

**Figure 3:**
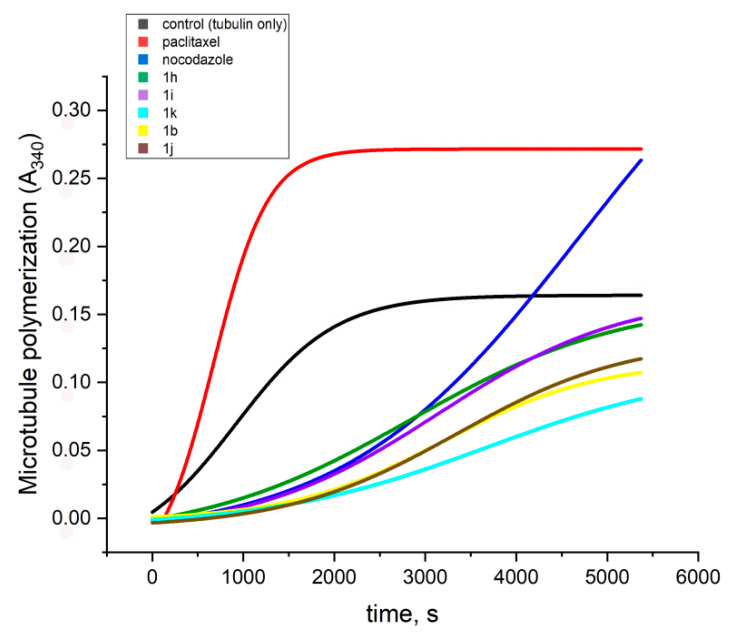
Porcine brain tubulin (final 70 µM) in presence of PB-GTP buffer, 10 µM paclitaxel, 10 µM nocodazole and 10 µM of the assayed compounds. Reaction was conducted in thermostated spectrophotometric chamber at 37 °C for 90 min. Turbidity was measured at 340 nm every 30 s.

It was found that compounds **1a–l** modulate the polymerization of tubulin at different extend depending on their molecular structure (Table 1). All of them, except for **1l**, elongated the nucleation phase and all slowed down the tubulin polymerization in a higher extend than nocodazole. The kinetic curves for the tubulin polymerization in the presence of some of the compounds exhibiting the strongest modulation effects as well as the reference paclitaxel and nocodazole, are depicted in Figure 3.

Depending on the measured lag time (time of nucleation) and initial rates of the polymerization phase, the compounds within the series could be divided into three subgroups. The first of them encompasses the derivatives containing only hydroxy substituents in the phenyl ring **1a–d**: they elongated the nucleation phase up to 1000–1461 s and slowed down the polymerization to 4.9–13.4 a.u.s^−1^ × 10^−6^. In comparison, the spontaneous tubulin polymerization occurs at initial rate of 75.3 a.u.s^−1^ × 10^−6^ and the modulating effect of nocodazole is related to lowering of the initial rate to 52 a.u.s^−1^ × 10^−6^. The second subgroup among the studied compounds—those containing only methoxy substituents in the phenyl ring **1f–i**, showed shorter lag times (807–1090 s) and slightly higher initial rates of polymerization (10.7–23.1 a.u.s^−1^ × 10^−6^). The third subgroup—including the derivatives containing both hydroxy and methoxy substituents in the phenyl ring **1j–l**, showed intermediate values of the two parameters: lag times of 1200–1295 s and initial rates of polymerization of 9.7–16.9 a.u.s^−1^ × 10^−6^. In the case of **1l** no lag phase was observed. 

The obtained results imply that merging the benzimidazole core with a phenyl hydrazone moiety provides an efficient scaffold for the design of tubulin polymerization inhibitors. Moreover, the hydroxy substituents in the phenyl ring ameliorate further the inhibition effect. 

**Figure 4:**
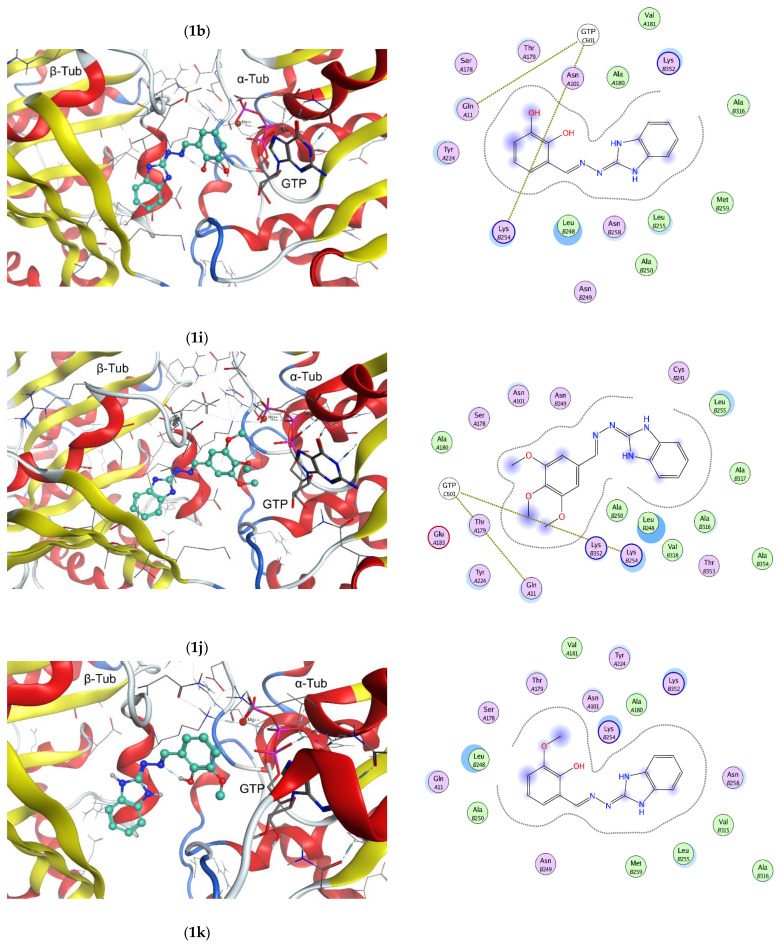

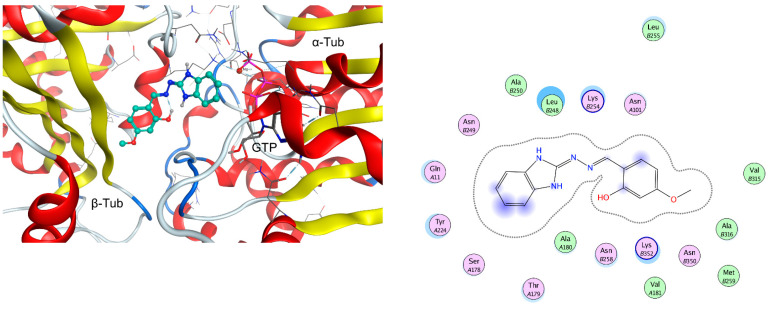
Three-dimensional (3D) representation of the interactions of ligands **1b**, **1i**, **1j** and **1k** at the colchicine binding site of the tubulin dimer (left panel) and interaction maps (right panel); the following colors were used for representation of the different components: proximity contour—black dotted line; polar amino acids—pink; lipophilic amino acids—green; basic amino acids—blue; acidic amino acids—red; hydrogen bond interactions—blue arrows, lipophilic interactions—green dotted lines. Docked structures are depicted by balls and sticks, GTP—by sticks. In the 3D representation of the interactions the α-tubulin subunit is positioned on the right side of the picture, the β-tubulin subunit—on the left side.

The possible binding modes of the 1H-benzimidazol-2-yl hydrazones with tubulin were explored by molecular docking at the colchicine binding site of a complex of αβ-tubulin with colchicine and vinblastine, PDB ID: 1Z2B [26]. Molecular docking study was performed by using MOE program package [27]. The interactions of selected ligands—**1b**, **1i**, **1j** and **1k** with the tubulin dimer are illustrated in Figure 4. The positions of the ligands in the active pocket of colchicine are shown on the left panel of the figure. The α-tubulin subunit is visible on the right side of the picture, while the β-tubulin subunit—on the left side. The guanosine-5′-triphosphate (GTP) in the α-tubulin subunit is depicted by sticks. The docked structures are located near the intra-dimer interface between the α- and β-tubulin subunits and depicted by balls and sticks. The interaction maps are shown on the right panel of Figure 4.

Three of the illustrated compounds (**1b**, **1i** and **1j**) are accommodated in the active pocket with their benzimidazole ring oriented towards the hydrophobic amino acid residues Leu248, Ala250, Leu255, Met259, Val315, Ala316 and Val318 of β-tubulin. The phenyl moieties of the compounds are oriented towards the polar amino acid residues Asn 101, Glu183 and Tyr 224 of α-tubulin and Asn249 of β-tubulin. The phenyl rings are in close proximity of GTP and can interact with the polar amino acid residues Ser178 and Thr179 of α-tubulin T5 loop; Gln11 in T1 loop of α-tubulin and Lys254 within the H8 helix of β-tubulin, respectively. The fourth compound—**1k** lays in an opposite manner: with its benzimidazole core facing the GTP zone and the hydroxy/methoxy substituted phenyl ring towards the β-tubulin cavity. As Figure 4 shows, the binding of compounds **1b**, **1i**, **1j** and **1k** to tubulin is expected to be driven by Van der Waals interactions. 

Based on the reported X-ray structures of αβ-tubulin in complex with known inhibitors binding to the colchicine site, it was established that the binding of the ligands leads to a switch of the T7 loop. In the course of microtubule assembly, when the tubulin dimers should change their “curved” conformation to a “straight” structure in order to assemble, the intermediate domain of both α- and β-tubulin subunit move. In this conformational change strands S8 and S9 move closer to helix H8, and in the same time the colchicine site is contracted in order to enable translation of H7 in the right position. The binding of a ligand to the colchicine site prevents the “curved-to-straight” conformational change and thus inhibits the microtubule assembly [28,29,30]. Having in mind the interactions of the 1H-benzimidazol-2-yl hydrazones with the αβ-tubulin dimer retrieved from the docking study, it could be anticipated that the mechanism of tubulin polymerization inhibition of compound **1a-l** relays namely on hampered “curved-to-straight” conformational change.

### 2.3. In Vitro Cytotoxicity

A standard colorimetric MTT assay was used to evaluate the anticancer activity of the newly synthesized 1H-benzimidazol-2-yl hydrazone derivatives against human malignant cell lines of different histological origin, namely MCF-7 (ER-positive breast adenocarcinoma) and AR-230 (BCR-ABL positive chronic myeloid leukemia), using podophyllotoxin as a reference drug. For each compound, the half-inhibitory concentrations (IC_50_) were estimated based on the derived “dose–response” relationships following 72h exposure time. In addition, their cytotoxicity was measured against normal fibroblast cells 3T3 (mouse embryo fibroblasts) and CCL-1 (murine fibroblasts) and respective cancer selectivity indices (CSI) were calculated (Table 2).

**Figure 5:**
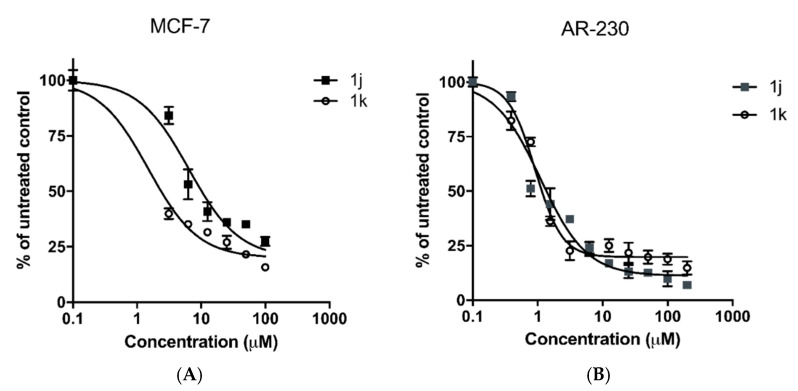
Cell viability of MCF-7 (panel **A**) and AR-230 (panel **B**) cell lines following 72h exposure to the lead compounds **1j** and **1k** as assessed by MTT-dye reduction assay. All groups are compared to an untreated control. Statistical analysis is performed by ANOVA one-way test and Tukey post test.

As indicated by the presented experimental data, the newly synthesized benzimidazole compounds demonstrate a marked antineoplastic activity in low micromolar concentrations in both screened in vitro tumor models. The leading structures of the series include the trisubstituted benzimidazole derivative **1i**; as well as the disubstituted positional isomers **1j** and **1k**. Their half-inhibitory concentrations (IC_50_) in both tumor cell lines are similar to those of the reference drug, but in contrast, the benzimidazole derivatives are distinguished by high selectivity with regard to malignantly transformed adenocarcinoma and leukemic cells compared to the normal 3T3/CCL-1 cell culture. The determined CSIs for the three compounds were strictly > 50 in respect to MCF-7 tumor cell line, whereby against AR-230 myeloid cells the highest tumor selectivity exhibited the *ortho*- disubstituted isomer **1j** (CSI ≈ 363) followed by **1k** (CSI ≈ 113). The cell viability of MCF-7 and AR-230 cell lines following 72h exposure to compounds **1j** and **1k** are illustrated in Figure 5. 

A certain “structure–activity” relationship was also observed between the analogues in the series, according to which their activity decreases in the order: **1k**, **1j** (*meta*/*ortho* heterosubstituted derivatives with both -OH and -OCH_3_ groups in the phenyl ring) > **1g**, **1h** (disubstituted analogues with two -OCH_3_ methoxy groups in *meta*-position) > **1a**, **1f** (monosubstituted benzimidazoles with a hydroxy- or methoxy-group in the phenyl core) > **1b**, **1d** (dihydroxy-substituted phenyl ring). Favorably, the cancer selectivity of the compounds follows the same pattern. Based on this, it can be concluded that the two substituents -OH and -OCH_3_ are key pharmacophore elements and their mutual presence in the structure of the experimental compounds synergistically improves their antitumor activity and cancer selectivity.

The cytotoxicity data for two of the most potent compounds in the series **1j** and **1k** are consistent with the results from the tubulin assay presented in Section 2.2. Thereby, both heterosubstituted analogues had a great impact on the rates of tubulin polymerization increasing the lag time of the process to more than 20 min (Table 1). Interestingly, the most efficient modulators of tubulin kinetics (lag time of 1400 s), the **1b** and **1d** benzimidazoles lacking an OCH_3_ group, displayed the poorest cytotoxicity and cancer selectivity in the series. Considering this, alternative additional mechanistic properties are likely to be involved in the antitumor effects of the experimental compounds.

Given their benzimidazole structure and the existing literature data on the biological activity of benzimidazole derivatives, a further assumption can be made about the molecular basis of their superior cytotoxicity against the MCF-7 and AR-230 cell lines. For example, hybrid compounds carrying a benzimidazole moiety have been reported to modulate estrogen receptors (ER-α) [31,32,33,34] which are overly expressed in the hormone-sensitive breast cancer model used in our study (MCF-7). Structural similarities of the benzimidazole nucleus to nonsteroidal triazole aromatase inhibitors makes it a suitable pharmacophore in targeting the enzyme involved in the final stages of estrogen biosynthesis. Recently, Çevik et al. synthesized novel benzimidazole-triazolothiadiazine hybrids and demonstrated their notable anti-aromatase activity in MCF-7 cells [35].

The leukemic AR-230 cell line is bcr-abl+ (positive for the fusion bcr-abl tyrosine kinase gene, formed in the Philadelphia chromosome rearrangement). Targeting the encoded cytoplasmic tyrosine kinase bcr-abl that is constitutively activated in CML has been extensively studied to decelerate myeloid proliferation and overcome resistance issues. Numerous compounds with a diverse or related to the prototype drug imatinib structure have been described to inhibit the enzyme, including benzimidazoles. In particular, Hong and co-workers have synthesized a novel series of benzimidazole/benzothiazole derivatives and reported them to act as potent high-affinity inhibitors even of the T315I mutant bcr-Abl at low nanomolar concentrations [36].

### 2.4. Radical Scavenging Activity

**Figure 6:**
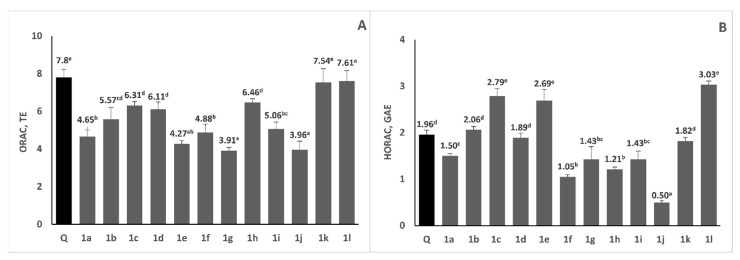
Antioxidant activity of compounds **1a–l** measured by ORAC (panel **A**) and HORAC (panel **B**) assays. Quercetin (Q) was used as a reference compound. Results are presented as mean values ± standard deviation (SD). There are no significant differences among values marked with the same superscript letters (*p* < 0.05).

In our previous study, it was revealed that some of the investigated 1H-benzimidazole-2-yl hydrazones reveal strong antiradical activity against the stable DPPH and ABTS radicals [25]. In the current study, we further investigated their antioxidant properties, applying two additional assays—ORAC and HORAC. Results for oxygen radical absorbance capacity and hydroxyl radical averting capacity of the studied 1H-benzimidazole-2-yl hydrazones are presented on Figure 6. ORAC measures the ability of antioxidants to scavenge physiologically relevant peroxyl radicals. Initially it was thought that it was carried out solely via HAT mechanism [37]; however, it was later proposed that both SPLET and SETPL mechanisms are competitive and more favorable than the HAT mechanism [38]. Tested compounds exerted moderate to high ability to scavenge peroxyl radicals, as reflected by their ORAC values, which ranged from 3.91 to 7.61 TE. Compounds **1k** and **1l** revealed very high ORAC, which was comparable to that of the strong chain-braking antioxidant quercetin, used as a reference compound. Interestingly, compound **1k** showed marked antineoplastic activity in the used in vitro tumor models, thus combining both potent antitumor and antioxidant properties. It is known that several structural features, including the number and the position of hydroxy groups influence peroxyl radical scavenging activity of antioxidants. From our results, it is evident that even without OH groups (compounds **1g** and **1i**), only due to the presence of N-bonded hydrogen atoms, the investigated 1H-benzimidazole-2-yl hydrazones reveal moderate ORAC values, comparable to those of some phenolic antioxidants, such as ferulic, p-coumaric acids [39], caffeic and chlorogenic acids [37]. One would expect that the presence of OH groups in phenyl ring, especially in *ortho*-position, would result in elevated ORAC values. However, the hydrazone **1l** containing hydroxy group on *metha*-position and methoxy group on *para*-position has the highest ORAC value—7.61 TE. On the contrary, the presence of OH group on 2-position lowered the oxygen radical absorbance capacity of the compounds. According to the literature data, benzimidazolyl-2-hydrazones could interconvert between two tautomeric forms (amino and imino) and the imino form strongly prevails in all compounds containing hydroxy group in *ortho*-position of the phenyl ring, otherwise the amino form is the favorable one [25]. This is accompanied by the formation of hydrogen bond between the OH group on 2-position and the N-atom, which probably would block that OH group and its ability to donate hydrogen atom.

The HORAC assay measures the metal-chelating activity of antioxidants in the conditions of Fenton-like reactions employing a Co(II) complex and hence the protecting ability against formation of hydroxyl radical. HORAC values of various phenolic compounds are related to their metal chelating properties and molecules that can chelate metals show significant HORAC values [40]. According to the HORAC assays, the antioxidant capacity ranged between 0.50 and 3.03 GAE. Compounds **1e**, **1c** and **1l** revealed higher hydroxyl radical averting capacity (2.79 GAE, 2.69 GAE and 3.03 GAE, respectively) than the used reference compound quercetin, which means that these compounds are strong metal chelators. Another group of compounds (**1b**, **1d** and **1k**) revealed moderate HORAC value around 2 GAE, which is comparable to that of quercetin. When comparing the structures of the investigated compounds, it was revealed that besides the hydrazone **1l** (containing hydroxy group on *metha*-position and methoxy group on para-position), the presence of two or three hydroxy groups in the phenyl moiety led to benzimidazole derivatives (**1b**, **1c**, **1d** and **1e**) with higher HORAC values. These HORAC values are higher or comparable to those of well-known phenolic antioxidants, such as protocatechuic acid, gallic acid, caffeic acid, chlorogenic acid, ferulic acid and catechin [40]. Structures containing only methoxy moieties in the phenyl ring (**1f**, **1g**, **1h** and **1i**) reveal low HORAC values. To our knowledge, the current study is the first one to report ORAC and HORAC activities of 1H-benzimidazole-2-yl hydrazones. These results could be employed in the development of new drugs with antineoplastic and antioxidant actions, expressed by either direct radical scavenging or metal chelating. 

### 2.5. Prediction of the Physico-Chemical Properties and Drug-Likeness

Study of the toxicity potential is an important step for the development of new drug candidates. Since the traditional pre-clinical analysis required a lot of time, funds and endanger animal life, in the last few decades several computational approaches were developed for prediction of drug toxicity [41]. In order to exert their therapeutic effect, the molecules have to meet certain requirements such as oral availability, transportation in the cells, ability to interact with biological targets. Therefore, several ADME (absorption, distribution, metabolism and excretion) properties were estimated using the Molinspiration tool [42], the method Brain Or IntestinaL EstimateD (BOILED-Egg) [43] and the bioavailability radar of the program SwissADME [44]. The overall drug likeness was characterized by the combined parameters within the methodology developed by Lipinski and co-authors, known as the “Rule of Five” [45]. The data from the Molinspiration prediction are summarized in Table 3. The results from the SwissADME prediction are shown in Figures S1–S12 and Tables S2–S13 in the Supplementary Material.

Molinspiration tool predicts logP values below 4 for all synthesized compounds **1a–l**, which suggests good absorption and bioavailability (Table 3). The topological polar surface area (TPSA), which is also used as indicator of the bioavailability, blood–brain penetration and intestinal absorption [46,47], is below 140 Å^2^ for all hydrazones **1a–l**, so they are expected to exhibit good intestinal absorption. Compounds **1f, 1g, 1h** and **1a** with TPSA values 62–73 Å^2^ approach the limit for blood–brain barrier penetration (below 60 Å^2^). The blood–brain barrier penetration characterizes the ability of microcirculation in the central nervous system and it overwhelms the penetration of large molecules and more than 98% of the smaller drugs [48]. The flexibility of the molecule could affect the oral bioavailability and the interaction with the biological targets [46]. Hydrazones **1a–d**, **1g–h** and **1j–l** show low number of rotatable bonds (N_rotb_)—6 or less (Table 3) consistent with sufficient oral bioavailability. All 1H-benzimidazole-2-yl hydrazones show good hydrogen-bonding capacity with 5–7 H-bond acceptors and 2 to 5 H-bond donors (Table 3) which is beneficial for efficient binding to biological targets. In line with the Molinspiration tool, SwissADME predicts high intestinal absorption for all compounds as well as good blood–brain barrier penetration for compounds **1a** and **1f–h**. The results regarding the TPSA parameter from the program SwissADME are in good correlation with the data from Molinspiration. For the solubility of compounds **1a–l** in water the SwissADME predicted values from −3.43 to −4.04, i.e., they would be soluble enough to act as orally administered pharmaceutical agents and deliver a sufficient amount of the active ingredient [49,50]. According to the algorithm, values below −10 correspond to a ligand insoluble in water, from −10 to −6: slightly soluble, from −6 to −4: moderately soluble, from −4 to −2: soluble, from −2 to 0: very soluble and above 0: highly soluble [44]. The coefficient of skin permeability (log Kp) was estimated by SwissADME tool based on a linear correlation between the size of the molecule and its lipophilicity. The more negative the log Kp value (with Kp in cm.s^−1^), the less permeable is the molecule through the skin [44]. For the studied compounds **1a–l** log Kp varies in the interval from −5.50 to −6.35. The hydrazone **1e** is the least permeable through the skin with log Kp = −6.35. Summarizing the above parameters, it could be concluded that none of the 1H-benzimidazole-2-yl hydrazones **1a–l** violates the Lipinski’s rule. The results from the SwissADME radar of bioavailability also support that the compounds meet all requirements for drug likeness, with the sole exception of the values for saturation (carbon atoms in sp^3^-hybridization).

The effect on pharmacokinetic proteins such as P-glycoprotein (P-gp) and cytochromes P450 (CYP) was also evaluated by SwissADME. The main function of P-gp is to protect the central nervous system from the influence of xenobiotics so they could be excreted in some tumor cells thus leading to drug resistance [51]. On the other hand, CYP isoenzymes are involved in the drug elimination through metabolic biotransformation [44]. The inhibition of the isoenzymes is certainly one of the main causes of drug interactions leading to toxic or other side effects [52] due to the lower rate of elimination of the drug and its accumulation in the body [53]. The results showed that none of the hydrazones is expected to be substrate of the P-gp protein and they would not be eliminated from the CNS. Most of the studied ligands are expected to inhibit the CYP1A2 isoform while compounds **1f–j** and **1l** might be inhibitors of both CYP2C19 and CYP2D6 enzymes. 

A preliminary estimation of the toxicological properties of compounds **1a–l** was performed by OSIRIS Property explorer [54] and summarized in Table S1 in the Supplementary Material. The program estimates the mutagenic, irritant, tumorigenic and reproductive risk. All of the synthesized hydrazones are expected to show low tumorigenic risk. Most of the compounds are expected to exhibit low mutagenic risk as well. Only the presence of a hydroxy or methoxy group at the metha-position in the phenyl ring of hydrazones **1b** and **1j** might lead to an increased mutagenic risk. The expected irritant and reproductive risk for most of the compounds is also low. Medium irritant risk is expected only for compound **1j** and a higher reproductive risk—for its positional isomer **1k**, respectively.

## 3. Materials and Methods

### 3.1. In Vitro Tubulin Polymerization Assay

The effect of the studied compounds on the polymerization of purified porcine tubulin was monitored using a commercial assay kit (cat. number BK004P), purchased from Cytoskeleton, USA. Stock solutions of porcine brain tubulin solutions (4 mg.mL^−1^) were prepared immediately prior to use in general tubulin buffer (80 mM piperazine-N,N′-bis(2-ethanesulfonic acid, PIPES) pH 6.9, 2 mM MgCl_2_ and 0.5 mM (ethylene glycol-bis(β-aminoethyl ether)-N,N,N′,N′-tetraacetic acid, EGTA) containing 1 mM guanosine triphosphate (GTP) (G-PEM buffer). Stock solutions of Paclitaxel (100 µM), Noconazole (100 µM) or tested samples (100 µM) in G-PEM buffer containing 5% DMSO were prepared. 

The standard reaction mixture contains 95 μL of 4 mg.mL^−1^ porcine brain tubulin and 5 μL of standards, test samples or G-PEM buffer only (control). Then, the microplate with loaded reaction mixtures was incubated for 90 min. at 37 °C in a thermostated spectrophotometer chamber. The turbidity of the mixtures was measured at 340 nm every 30 s. The experiment was performed on SpectroStar Nano (BMG Labtech, Aylesbury, UK) equipped with software for kinetic measurements.

### 3.2. Molecular Docking 

Docking was performed by the Molecular Operating Environment (MOE) 2020 software package [27]. The model was built upon the structure of αβ-tubulin with the stathmin-like domain of the RB3 protein in complex with colchicine and vinblastine, obtained by XRD with an overall resolution of 4.10 Å resolution, PDB ID: 1Z2B [26]. The structure was protonated (pH 7.0, 300K, Salt 0.1 M/L) using the 3d protonation algorithm implemented in the MOE package. Different conformations of the studied compounds were included in the docking study accounting for amino and imino tautomeric forms, *E* and *Z* configuration of the azomethyne double bond, *s-cis* and *s-trans* arrangement of the substituents around the N-N bond, formation of intramolecular hydrogen bonds, etc. All conformations of all ligands were docked in the colchicine pocket using the Triangle Matcher algorithm for the initial placement of the structures, which returns up to 10 × 10^6^ poses of the ligand inside a pocket. These poses were scored by the London dG [MOE] function which estimates the free energy of the binding of the ligand from a given pose and consists of terms that estimate average gain/loss of rotational and translational entropy and loss of flexibility of the ligand. It measures the geometric imperfections of the hydrogen bonds and the desolvation energy of atoms. The best 50 poses for every ligand were further optimized with the Induced Fit methodology, using the MMFF94x force field/Born solvation model and optimization cutoff of 6Å from the ligand. The GBVI/WSA dG [27] was used as rescoring function and the best 30 poses were collected for further analysis.

### 3.3. Cytotoxicity Study 

The cell lines MCF-7, AR-231 and CCL-1 were obtained from the German Collection of Microorganisms and Cell Cultures. Cells were kept in a controlled environment—RPMI-1640 medium supplemented with 10% fetal bovine serum (FBS) and 2mM L-glutamine, at 37 °C in an incubator with 5% CO_2_ humidified atmosphere.

MTT-based colorimetric assay was used for evaluating the cell viability. The method is based on the reduction of the yellow tetrazolium compound MTT to water-insoluble, violet formazan crystals [55]. MCF-7, AR-231, and CCL-1 cells were seeded in 96-well, flatbottomed microplates (100 µL/well) at a density of 3 × 10^5^ cells per mL and allowed to grow for 24 h prior the exposure to the studied compounds. Cells were exposed to the tested agents for 72 h. After the exposure period MTT solution (10 mg.mL^−1^) aliquots (100 µL/well) were added to each well. The plates were further incubated for 4 h at 37 °C and the MTT-formazan crystals formed were dissolved through addition of 110 mL of 5% HCOOH in 2-propanol. The absorption was measured at 580 nm using a microplate reader (Labexim LMR-1).

Evaluation of the cytotoxic activity of some of the compounds was performed on 3T3 (mouse embryo fibroblasts) cell line. The cells were cultivated in Dulbecco′s Modified Eagle′s Medium (DMEM), containing 10% FBS, with supplements L-glutamine, sodium pyruvate, antibiotic and non-essential amino acids, and allowed to adhere overnight at 37 °C in an incubator with 5% CO_2_ humidified atmosphere. At the end of the incubation, the cells were treated with different concentrations of the compounds in a final volume of 100 μL/well in triplicate wells for each treatment for 24 h at 37 °C in a CO_2_ incubator. All studies were repeated at least twice. Control cells were grown in DMEM containing 10% FBS and allowed to stabilize overnight at 37 °C in a CO_2_ incubator and left without treatment.

### 3.4. Antioxidant Study

#### 3.4.1. Chemicals

2,2′-azobis (2-amidino-propane) dihydrochloride (AAPH), 6-hydroxy-2,5,7,8-tetramethylchroman-2-carboxylic acid (Trolox), fluorescein disodium salt, cobalt (II) fluoride tetrahydrate and gallic acid were obtained from Sigma–Aldrich (Steinheim, Germany). Picollinic acid was purchased from Fluka (Deisenhofen, Germany). All other solvents used were of analytical grade and obtained from local producers.

#### 3.4.2. Oxygen Radical Absorbance Capacity (ORAC)

ORAC was measured according to the method of Ou et al. [37]. Fluorescein was used as the fluorescent probe. The loss of fluorescence of fluorescein was an indication of the extent of fluorescein damage in its reaction with the peroxyl radicals. The protective effect of antioxidants was measured by assessing the area under the fluorescence decay curve (AUC) as compared to that of blank in which no antioxidant was present. Solutions of AAPH, fluorescein and Trolox were prepared in phosphate buffer (75 mM, pH 7.4). Reaction mixture (total volume 200 μL) contained fluorescein (170 μL, final concentration 5.36 × 10^−8^ M), AAPH (20 μL, final concentration 51.51 mM) and sample (10 μL). All 1H-benzimidazole-2-yl hydrazones were dissolved in acetone in concentration 5 × 10^−6^ M (final concentration 0.25 μM). Fluorescein solution and sample were incubated at 37 °C for 20 min and AAPH (dissolved in 37 °C buffer) was added. The mixture was shaken directly in FLUOstar Galaxy plate reader for 30 s before the initial fluorescence was measured. After that, the fluorescence readings were taken at the end of every cycle (1 min) after shaking. For the blank, 10 μL of phosphate buffer was used instead of sample. Antioxidant activity was expressed in Trolox equivalents. For defining the standard curve 10 μL of 3.13, 6.25, 12.5, 25 and 50 μM Trolox solutions (final concentrations 0.16, 0.31, 0.63, 1.25 and 2.50 μM, respectively) were used instead of sample. One ORAC unit is assigned to the net protection area, provided by 10 μL, 1 μM Trolox solution (final concentration—0.05 μM). Results were expressed as Trolox Equivalents (TE).

#### 3.4.3. Hydroxyl Radical Averting Capacity (HORAC)

HORAC was performed according to Ou et al. [40] and fluorescein was used as the fluorescent probe. Hydrogen peroxide solution of 0.55 M was prepared in distilled water. 4.6 mM Co(II) was prepared as follows: 15.7 mg of CoF_2_.4H_2_O and 20 mg of picolinic acid were dissolved in 20 mL of distilled water. Fluorescein—170 µL (60 nM, final concentration) and 10 µL of sample were incubated at 37 °C for 10 min directly in the FLUOstar plate reader. All 1H-benzimidazole-2-yl hydrazones were diluted in acetone in concentration 50 × 10^−6^ M (final concentration 2.5 μM). After incubation, 10 µL H_2_O_2_ (27.5 mM, final concentration) and 10 µL of Co(II) (230 µM final concentration) solutions were added subsequently. The initial fluorescence was measured after which the readings were taken every minute after shaking. For the blank sample, phosphate buffer instead of sample was used. 31.25, 62.5, 125, 250 and 500 µM gallic acid solutions (final concentrations: 1.56, 3.13, 6.25, 12.5 and 25 µM) in phosphate buffer (75 mM, pH = 7.4) were used for building the standard curve. The AUC were calculated as they were for the ORAC assay. One HORAC unit was assigned to the net protection area provided by 1 µM gallic acid (final concentration −0.05 μM) and the activity of the sample was expressed as Gallic Acid Equivalents (GAE).

ORAC and HORAC analyses were carried out on a FLUOstar Galaxy plate reader (BMG Labtechnology, Offenburg, Germany) with excitation wavelength = 485 nm and emission wavelength = 520 nm.

## 4. Conclusions

Most of the 1H-benismidazol-2-yl hydrazones studied exhibit pronounced concentration-dependent cytotoxic activity against cellular MCF-7 breast cancer and chronic myeloid leukemia cell lines AR-230. The leading structures of the series: the trimethoxy substituted derivative **5i**, the positional isomers **5j** and **5k** with hydroxy and methoxy groups in the phenyl rings were outlined based on their low micromolar IC_50_ concentrations and good selectivity against the tumor cells. The in vitro study of the effect of benzimidazole derivatives on tubulin polymerization as a putative mechanism of antineoplastic action showed that the compounds modulate polymerization of tubulin to varying extend, depending on their molecular structure. Most compounds prolong the nucleation phase and retard tubulin polymerization to a greater extent than nocodazole. Molecular docking study of the investigated compounds in the colchicine binding site provided an insight on the possible interactions with tubulin. The 1H-benisimidazol-2-yl hydrazones exerted moderate to high ability to scavenge peroxyl radicals and certain derivatives among them such as **5l** containing hydroxy group on *metha*-position and methoxy group on *para*-position, and **5b, 5c, 5d** and **5e**—with two/three hydroxy groups in the phenyl moiety, revealed HORAC values high or comparable to those of well-known phenolic antioxidants. Thus the 1H-benisimidazol-2-yl hydrazones with varied hydroxy/methoxy patterns in the phenyl fragments were recognized as new agents exhibiting promising combined antioxidant and antineoplastic action. The preliminary computational estimation of the physico-chemical parameters of the compounds suggested overall drug likeness, bioavailability and ability to pass through physiological barriers suitable for future applications. Despite the progress made in experimental research with 1H-benzimidazole-2-yl hydrazones as emerging anticancer agents, further studies are required to establish the exact molecular mechanisms of their broad spectrum of antitumor activity. The observed high selectivity in the cytotoxicity of the newly synthesized benzimidazole compounds against malignant cell populations of epithelial and leukemic origin makes them an attractive object for future biochemical and pharmacological studies to elucidate the molecular basis of their preferential antitumor effects.

## Figures and Tables

**Table 1:** In vitro effect on tubulin polymerization of 1*H*-benzimidazole-2-yl hydrazones **1a–1**.

Compound ^1^	Lag Time, s	Initial Rate of the Exponential Phase, Signal Response a.u.s^−1^ × 10^−6^
tubulin (spontaneous polymerization)	-	75.3
paclitaxel	151	167
nocodazole	935	52
**1a**	1390	8.77
**1b**	1461	13.4
**1c**	1190	4.9
**1d**	1407	13.3
**1f**	807	17.1
**1g**	1000	10.7
**1h**	1090	23.1
**1i**	988	20.6
**1j**	1200	16.9
**1k**	1295	9.7
**1l**	no lag phase	16

^1^ final concentration of all compounds and standards is 10 µM.

**Table 2:** In vitro cytotoxicity of 1H-benzimidazole-2-yl hydrazones 1**a-l** against cell lines of different origin.

Cell Line/Compound	IC_50_ (μM ± SD)			CSI_MCF-7_	CSI_AR230_
	MCF-7 ^1^	AR-230 ^2^	3T3 ^3^/ CCL-1 ^4^		
Podophyllotoxin	0.3 ± 0.06	0.47 ± 0.09	2.7 ^4^	≈9	≈5.7
**1a**	18.7 ± 1.1	21.2 ± 2.3	204 ± 5.1 ^3^	≈11	≈9.6
**1b**	64.5 ± 6.2	3.3 ± 0.3	61.1 ± 1.8 ^3^	≈1	≈18.4
**1d**	-	61.8 ± 5.7	112.8 ± 9.1 ^4^	-	≈1.8
**1f**	11.2 ± 1.3	19.4 ± 2.2	355 ± 5.3 ^3^	≈31.6	≈18.2
**1g**	7.9 ± 2.1	11.5 ± 1.0	261 ± 4.7 ^3^	≈33	≈22.6
**1h**	4.6 ± 0.7	5.9 ±0.9	335 ± 7.0 ^3^	≈72.8	≈56.7
**1i**	**1.2 ± 0.2**	**1.7 ± 0.3**	**71 ± 2.4 ^3^**	**≈59.1**	**≈41.7**
**1j**	**6.4 ± 1.2**	**1.1 ± 0.2**	**>400 ^4^**	**≈62.5**	**≈363.6**
**1k**	**1.5 ± 0.3**	**0.9 ± 0.1**	**101.8 ± 7.3 ^4^**	**≈67.8**	**≈113**
**1l**	27.6 ± 3.5	16.8 ± 3.4	134.7 ± 1.3^3^	≈4.8	≈8

MCF-7 ^1^: ER-positive breast adenocarcinoma; AR-230 ^2^: bcr-abl+ chronic myeloid leukemia; 3T3 ^3^: mouse embryo fibroblast normal cell line; CCL-1 ^4^: murine fibroblast normal cell line; CSI—cancer selectivity index = IC_50_(normal cell line)/IC_50_(malignant cell line).

**Table 3:** Calculated molecular properties of compounds **1a-l** for assessment of the drug-likeness.

Compound	logP ^a^	TPSA ^b^	N_atoms_^c^	MW ^d^	N_ON_^e^	N_OHNH_^f^	N_viol_^g^	N_rotb_^h^	Vol ^i^
**1a**	2.98	73.30	19	252.28	5	3	0	3	223.95
**1b**	2.28	93.53	20	268.28	6	4	0	3	231.97
**1c**	2.48	93.53	20	268.28	6	4	0	3	231.97
**1d**	2.07	93.53	20	268.28	6	4	0	3	231.97
**1e**	2.01	113.76	21	284.27	7	5	0	3	239.99
**1f**	3.10	62.31	20	266.30	5	2	0	4	241.48
**1g**	3.06	71.54	22	296.33	6	2	0	5	267.03
**1h**	3.08	71.54	22	296.33	6	2	0	5	267.03
**1i**	2.67	80.78	24	326.36	7	2	0	6	292.57
**1j**	2.59	82.54	21	282.30	6	3	0	4	249.50
**1k**	3.02	82.54	21	282.30	6	3	0	4	249.50
**1l**	2.38	82.54	21	282.30	6	3	0	4	249.50

^a^ Octanol–water partition coefficient, calculated by the methodology developed by Molinspiration; ^b^ Topological polar surface area calculated by the sum of the polar surface areas occupied by the oxygen and nitrogen atoms; ^c^ Number of nonhydrogen atoms; ^d^ Molecular weight; ^e^ Number of hydrogen-bond acceptors (O and N atoms); ^f^ Number of hydrogen-bond donors (OH and NH groups); ^g^ Number of ‘Rule of five’ violations; ^h^ Number of rotatable bonds; ^i^ Molecular volume.

## Data Availability

Data are available within the article.

## Supplementary Materials

The following supporting information can be downloaded at: https://www.mdpi.com/article/10.3390/molecules28010291/s1, Figures S1–S12: BOILED-Egg models and bioavailability radars predicted by SwissADME program; Table S1: Toxicological properties of 1H-benzimidazole-2-yl hydrazones predicted by OSIRIS property explorer; Tables S2–S13: Physico-chemical properties and drug-likeness of 1H-benzimidazole-2-yl hydrazones predicted by SwissADME program.
