# Supplementary material for: Modulation Effect on Tubulin Polymerization, Cytotoxicity and Antioxidant Activity of 1H-Benzimidazole-2-Yl Hydrazones
Source: Molecules. 2022 Dec 29;28(1):291. doi: 10.3390/molecules28010291 (PMC9822270; doi:10.3390/molecules28010291)
Supplement: Supplementary file 1 [file molecules-28-00291-s001.zip › molecules-2114304-supplementary.pdf]

Modulation effect on tubulin polymerization, cytotoxicity and antioxidant activity of 1H-benzimidazole-2-yl hydrazones

Maria Argirova[1], Maya Guncheva[1], Georgi Momekov[2], Emiliya Cherneva[1,2], Rositsa Mihaylova[2], Miroslav Rangelov[1], Nadezhda Todorova[3], Petko Denev[1], Kameliya Anichina[4], Anelia Mavrova[4], Denitsa Yancheva[2,*]

[1] Institute of Organic Chemistry with Centre of Phytochemistry, Bulgarian Academy of Sciences, Acad. G. Bonchev str. Bl. 9,1113 Sofia, Bulgaria
[2] Faculty of Pharmacy, Medical University of Sofia, 2 Dunav str., 1000, Sofia, Bulgaria
[3] Institute of Biodiversity and Ecosystem Research, Bulgarian Academy of Sciences, 2 Gagarin Str., 1113 Sofia, Bulgaria
[4] University of Chemical Technology and Metallurgy, 8 Kliment Ohridski Blvd., 1756 Sofia, Bulgaria
* Denitsa.Pantaleeva@orgchm.bas.bg, deni@orgchm.bas.bg (D.Y.);

Contents:

**Table S1.** Toxicological properties of 1*H*-benzimidazol-2-yl hydrazones **1a-l** predicted by OSIRIS Property Explorer	2
**Figures S1-S12.** BOILED-Egg model and bioavailability radar of compounds **1a-l** predicted by SwissADME program	3-14
**Tables S2-S13.** Physico-chemical properties and drug-likeness of 1H-benzimidazole-2-yl hydrazones **1a-l** predicted by SwissADME program	15-29

Table S1. Toxicological properties of 1*H*-benzimidazol-2-yl hydrazones **1a-l** predicted by OSIRIS Property Explorer

**Compd**		**Mutagenic risk**	**Tumorigenic risk**	**Irritant risk**	**Reproductivity risk**
**1a**	R_1=H; R_2= 2-OH	low	low	low	low
**1b**	R_1=H; R_2= 2,3-diOH	high	low	low	low
**1c**	R_1=H; R_2= 2,4-diOH	low	low	low	low
**1d**	R_1=H; R_2= 3,4-diOH	low	low	low	low
**1e**	R_1=H; R_2= 2,3,4-triOH	low	low	low	low
**1f**	R_1=H; R_2= 4-OCH_3	low	low	low	low
**1g**	R_1=H; R_2= 2,6-diOCH_3	low	low	low	low
**1h**	R_1=H; R_2= 3,5-diOCH_3	low	low	low	low
**1i**	R_1=H; R_2= 3,4,5-triOCH_3	low	low	low	low
**1j**	R_1=H; R_2=2-OH-3-OCH_3	high	low	medium	low
**1k**	R_1=H; R_2=2-OH-4-OCH_3	low	low	low	high
**1l**	R_1=H; R_2=3-OH-4-OCH_3	low	low	low	low

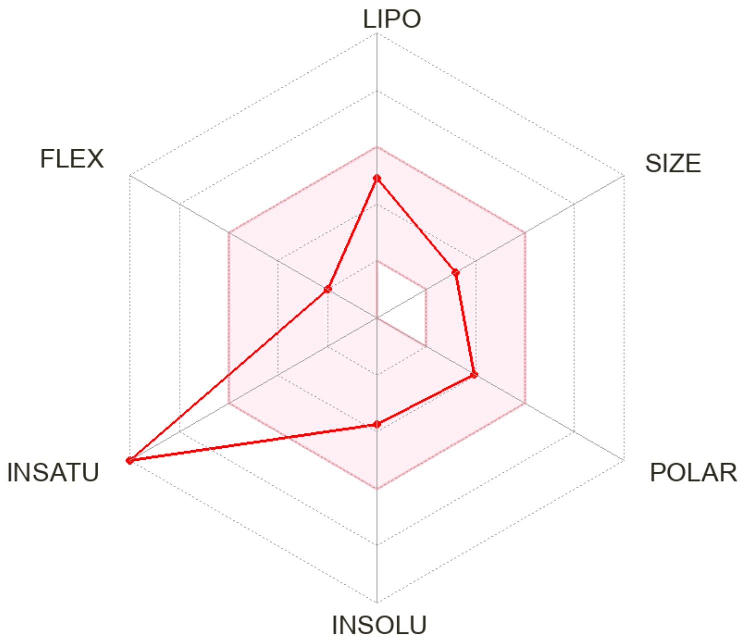

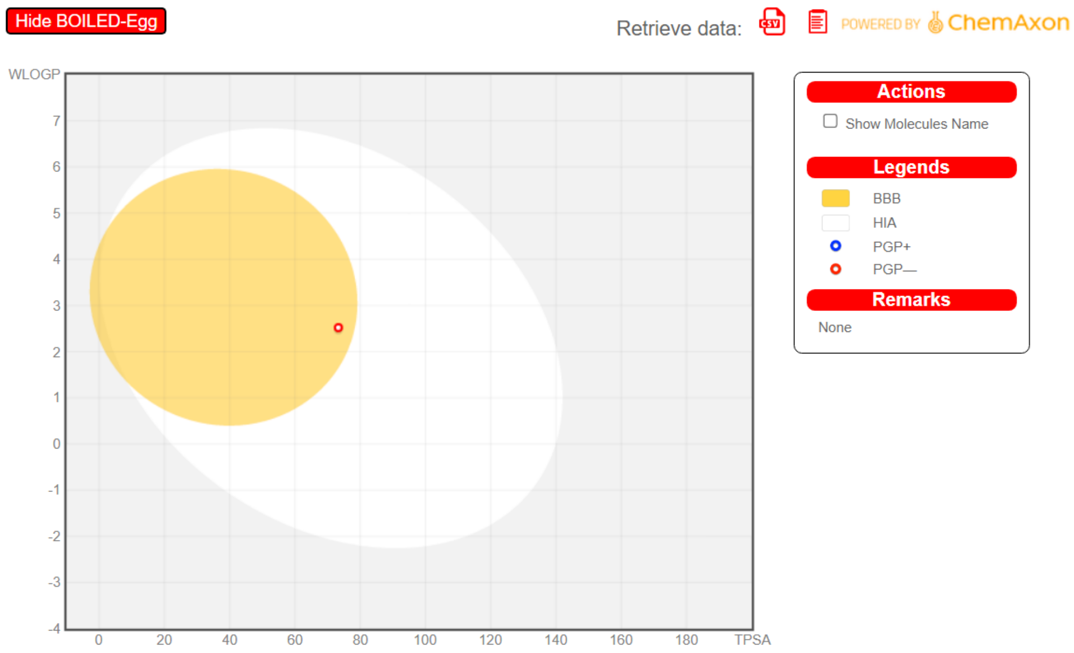

Figurw S1. Bioavailability radar and BOILED-Egg model of compound **1a** predicted by SwissADME program

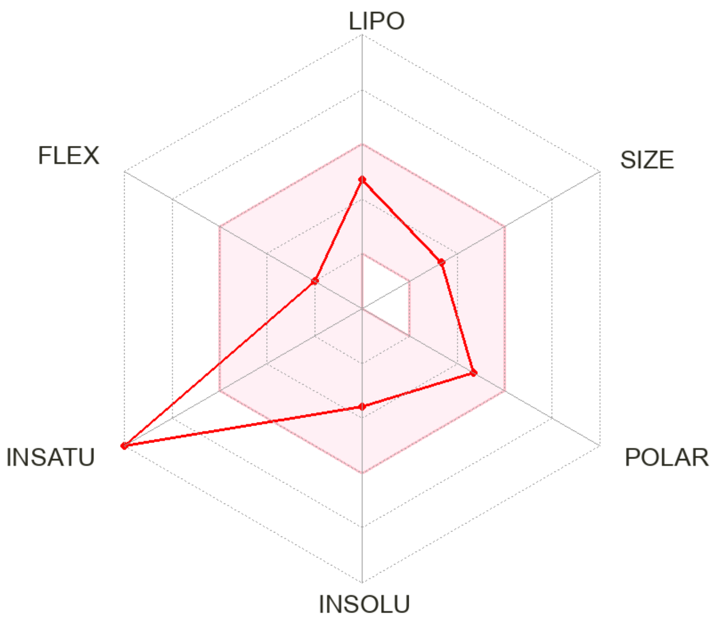

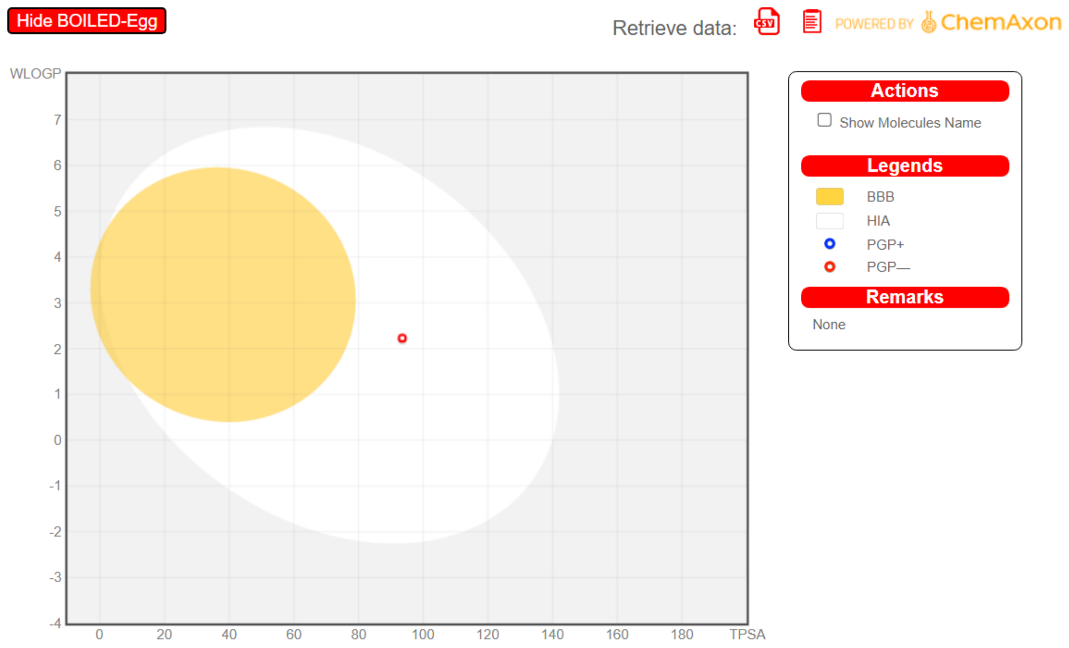

Figurw S2. Bioavailability radar and BOILED-Egg model of compound **1b** predicted by SwissADME program

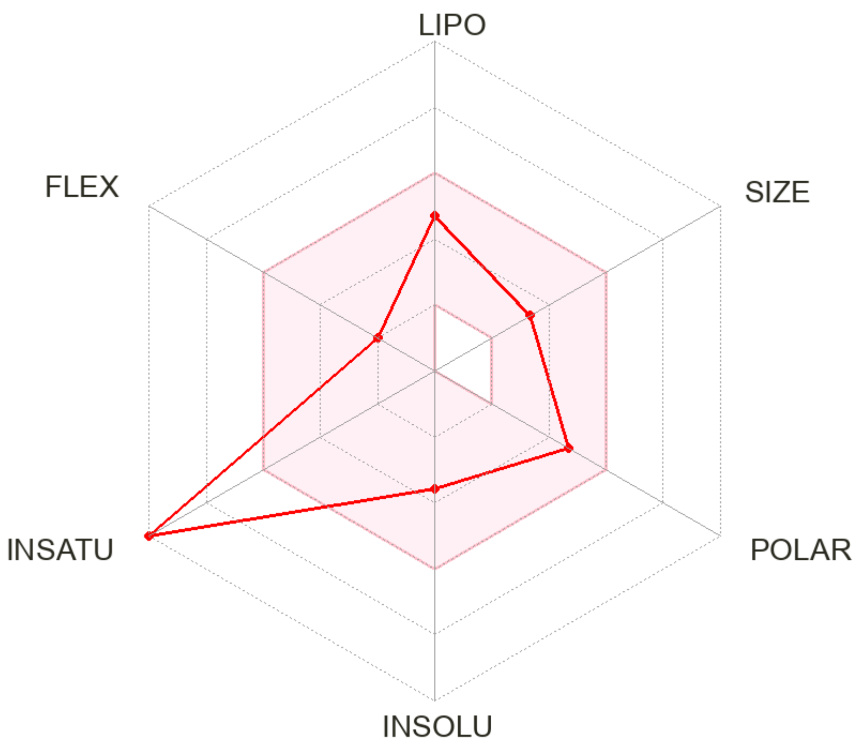

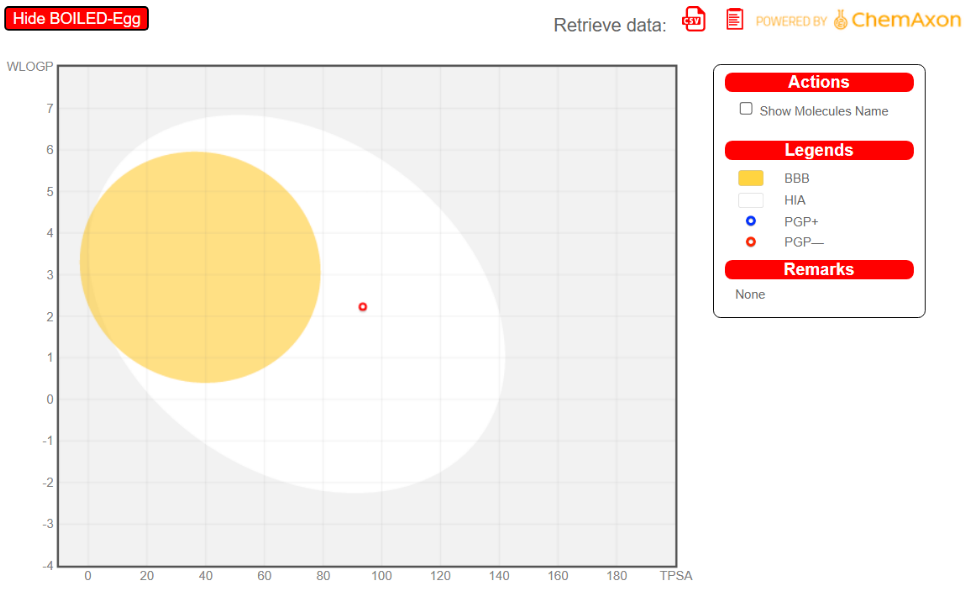

Figurw S3. Bioavailability radar and BOILED-Egg model of compound **1c** predicted by SwissADME program

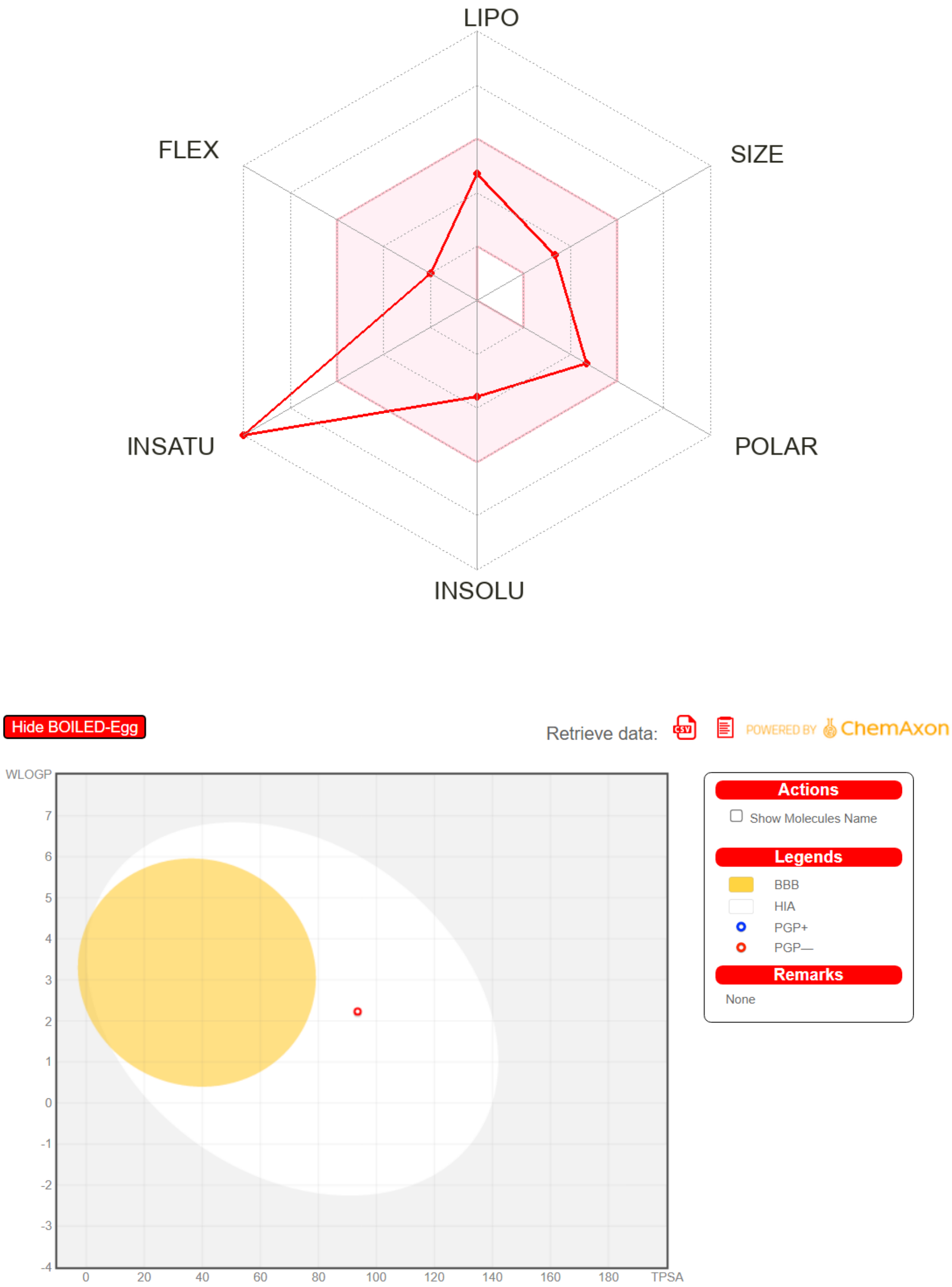

Figurw S4. Bioavailability radar and BOILED-Egg model of compound **1d** predicted by SwissADME program

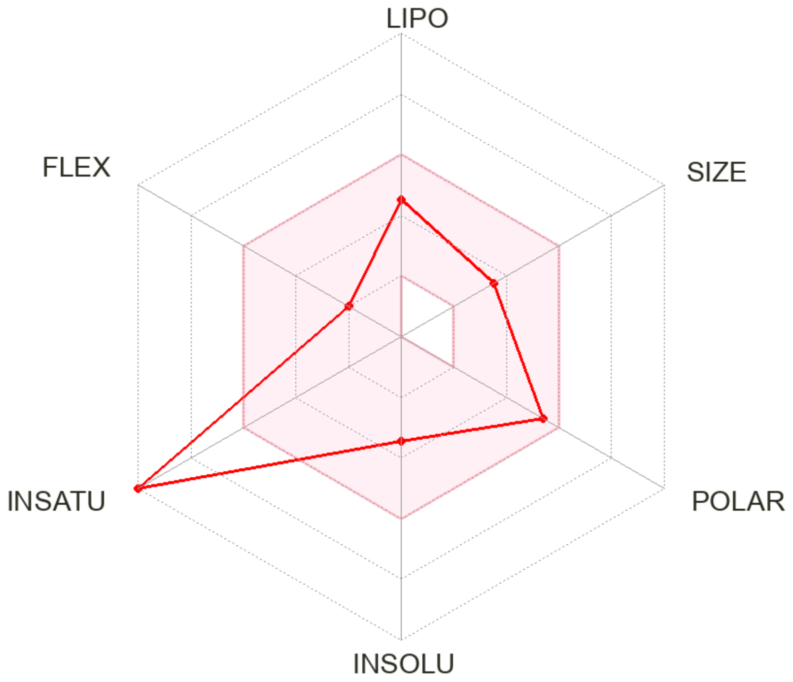

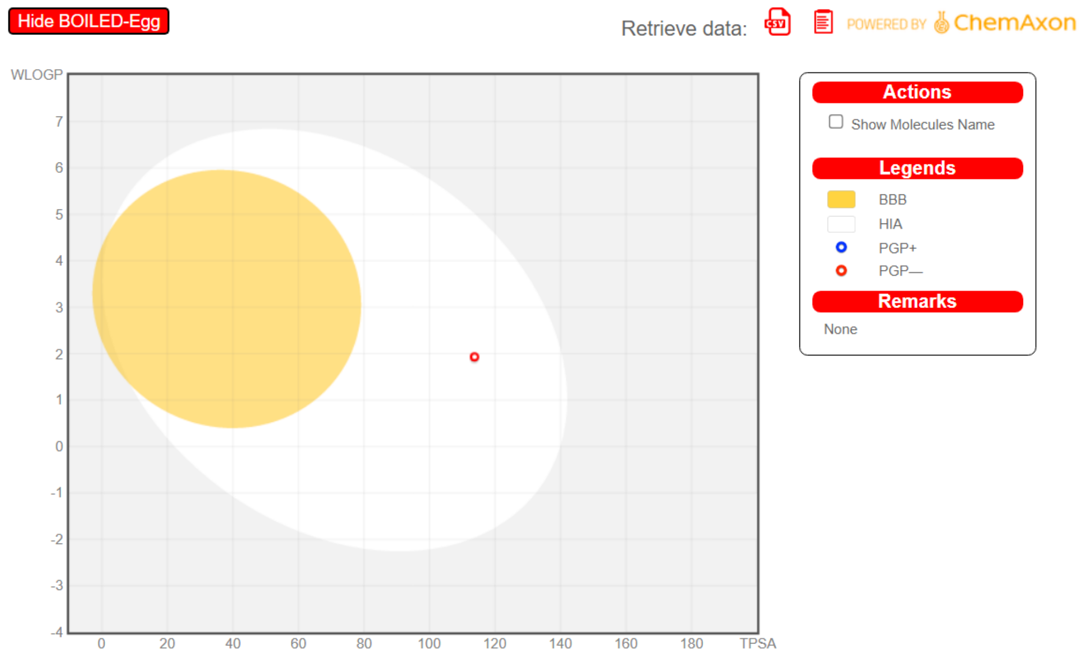

Figurw S5. Bioavailability radar and BOILED-Egg model of compound **1e** predicted by SwissADME program

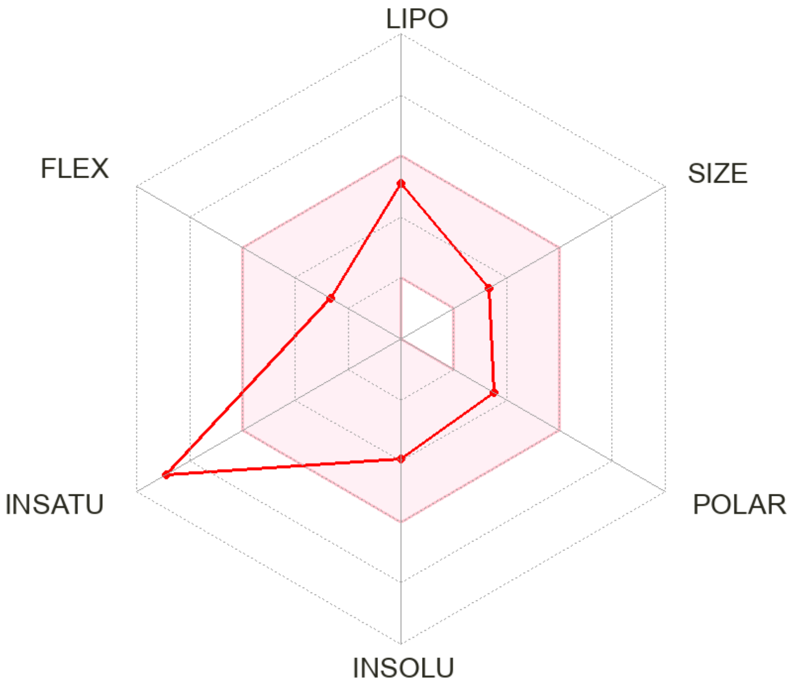

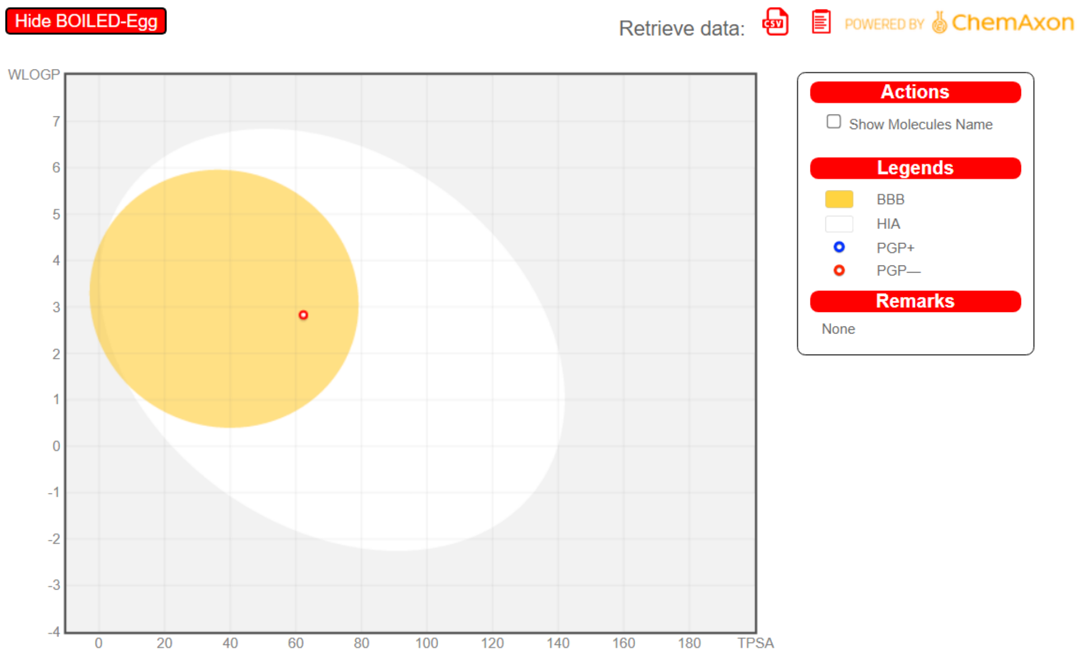

Figurw S6. Bioavailability radar and BOILED-Egg model of compound **1f** predicted by SwissADME program

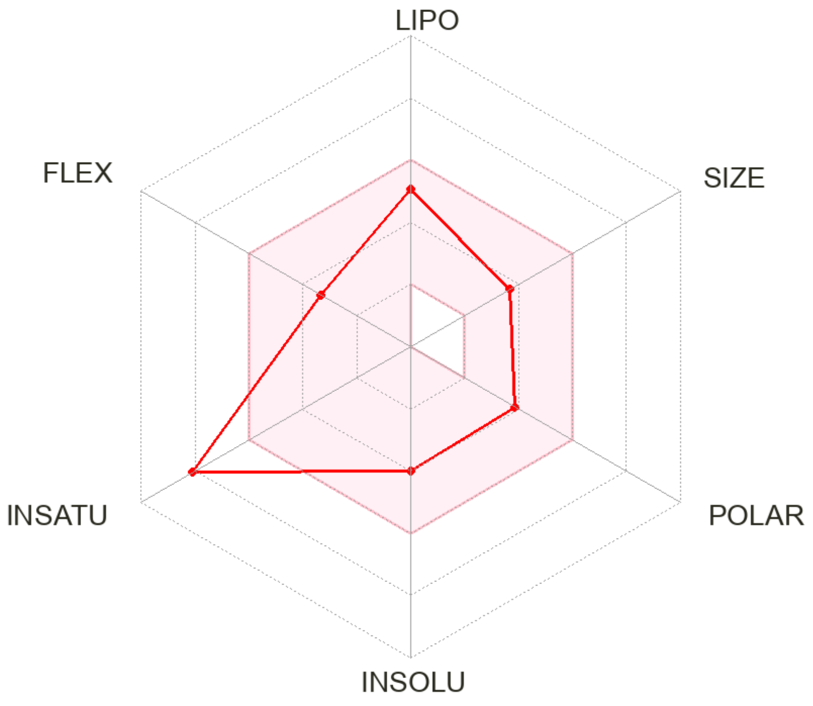

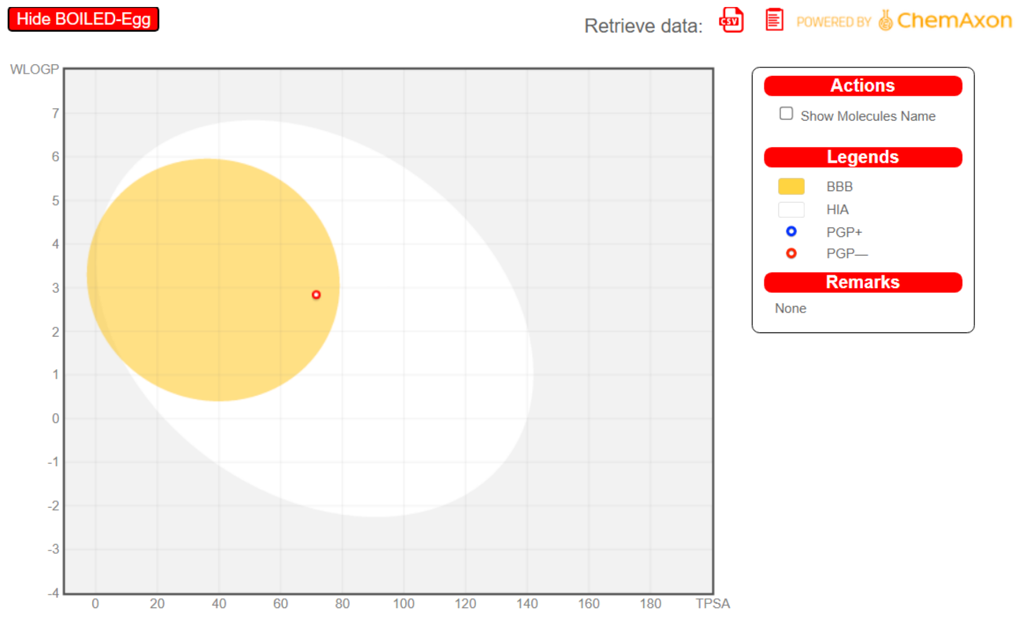

Figurw S7. Bioavailability radar and BOILED-Egg model of compound **1g** predicted by SwissADME program

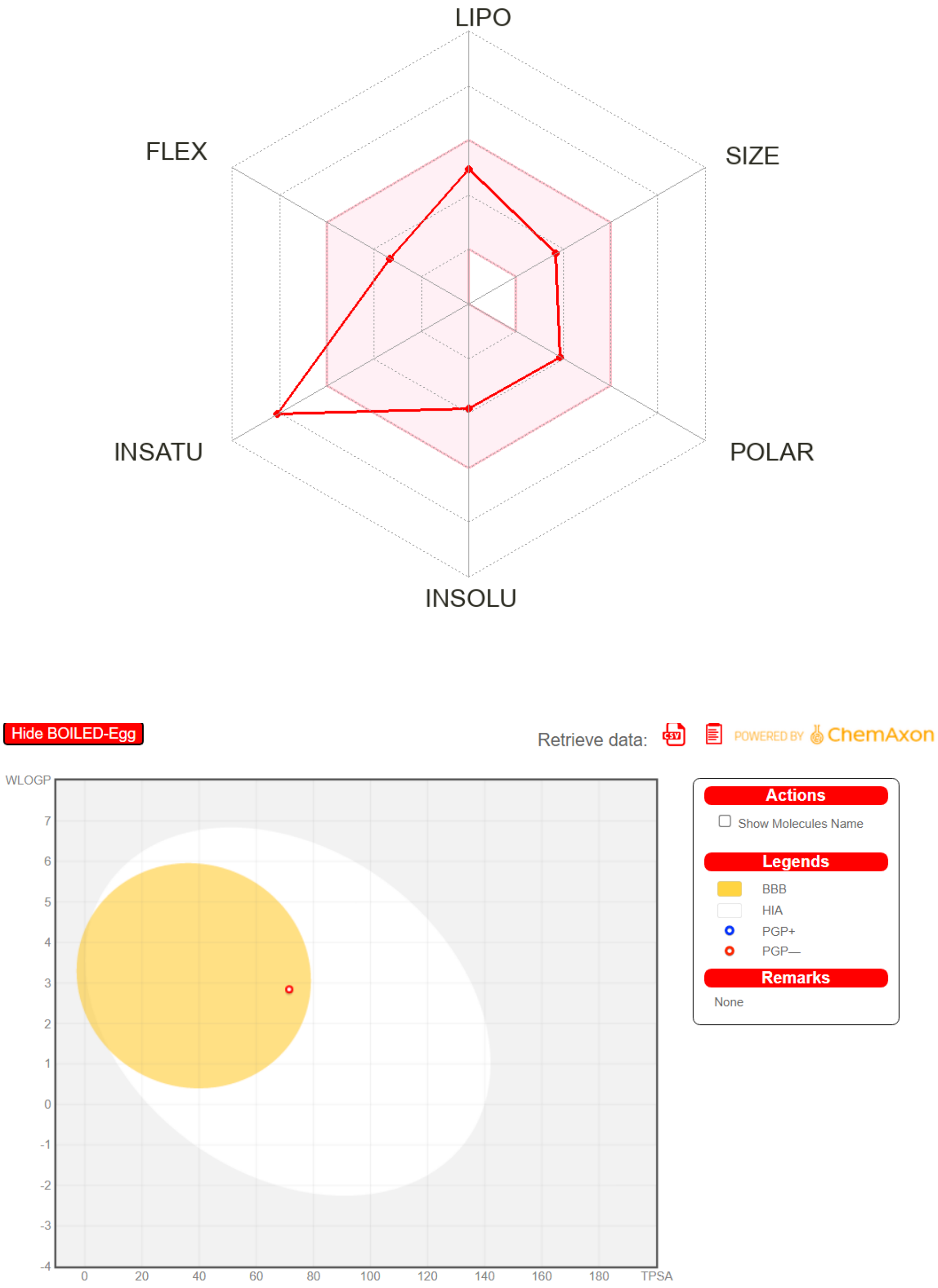

Figurw S8. Bioavailability radar and BOILED-Egg model of compound **1h** predicted by SwissADME program

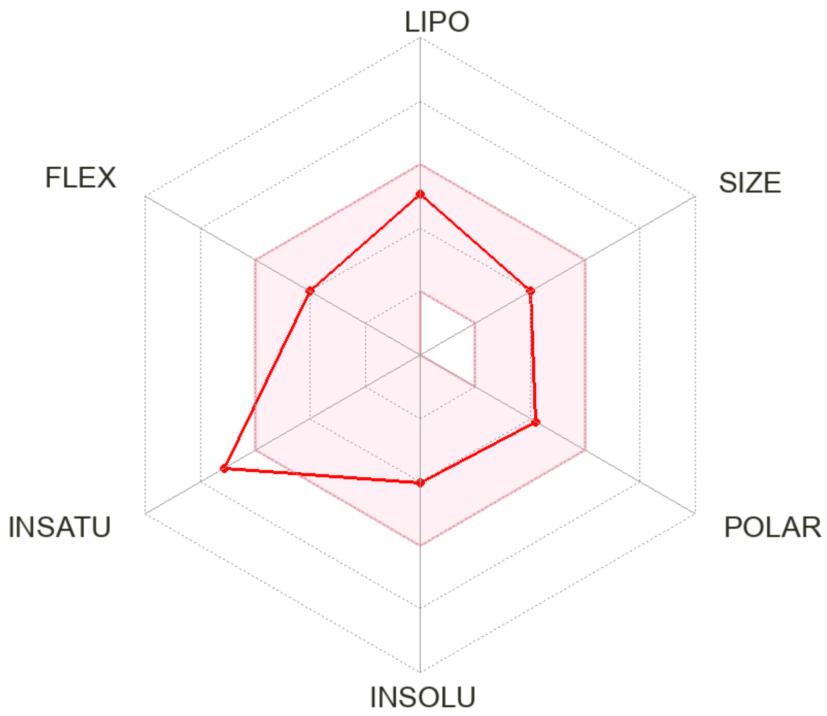

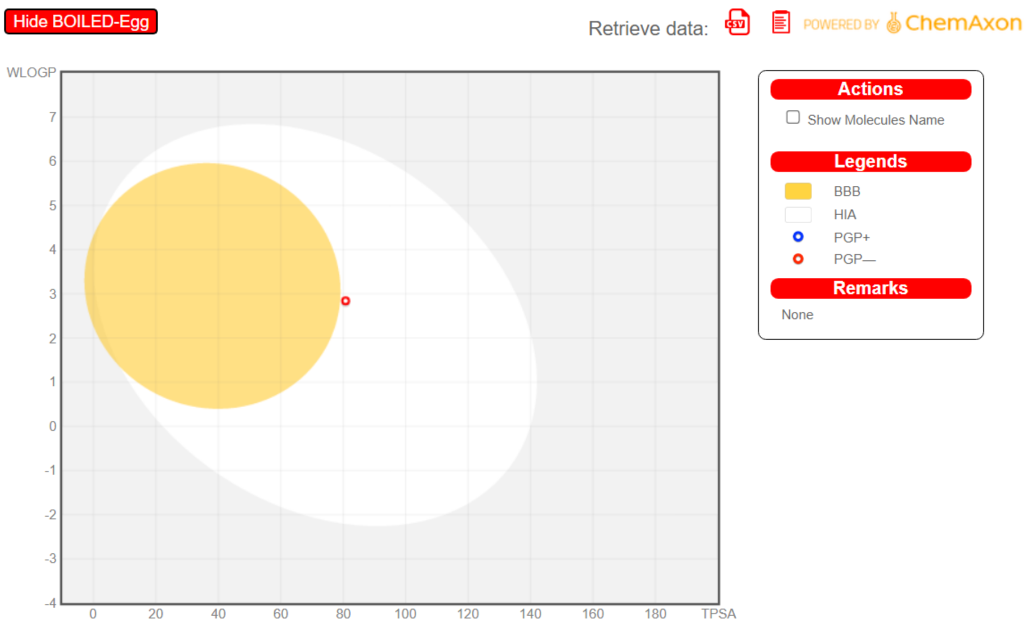

Figurw S9. Bioavailability radar and BOILED-Egg model of compound **1i** predicted by SwissADME program

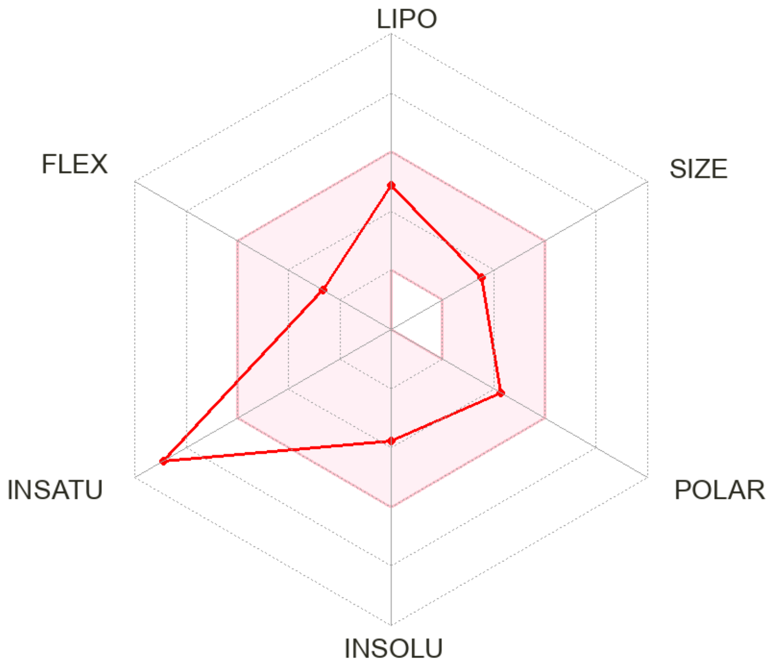

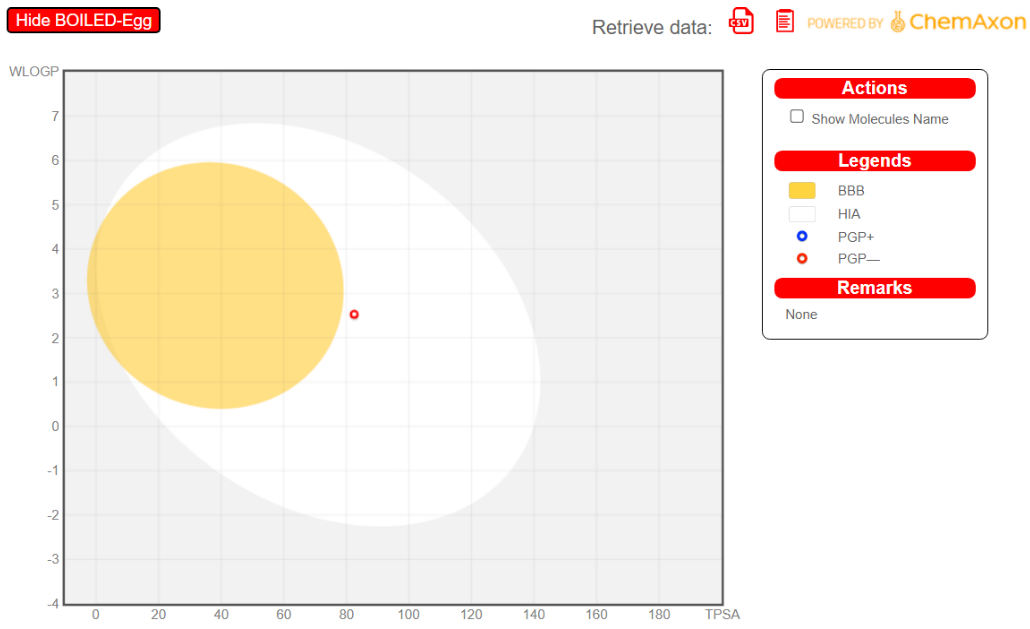

Figurw S10. Bioavailability radar and BOILED-Egg model of compound **1j** predicted by SwissADME program

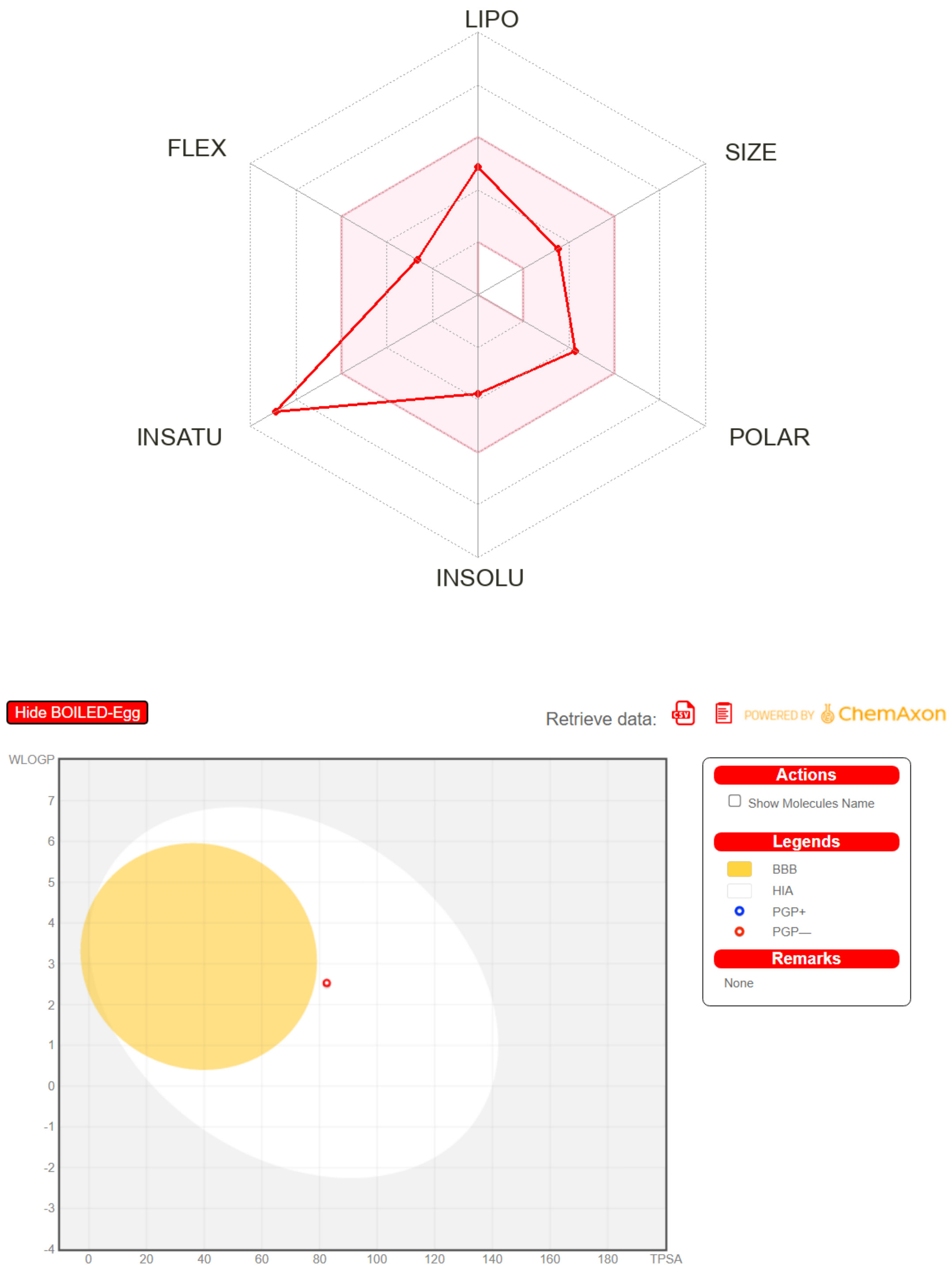

Figurw S11. Bioavailability radar and BOILED-Egg model of compound **1k** predicted by SwissADME program

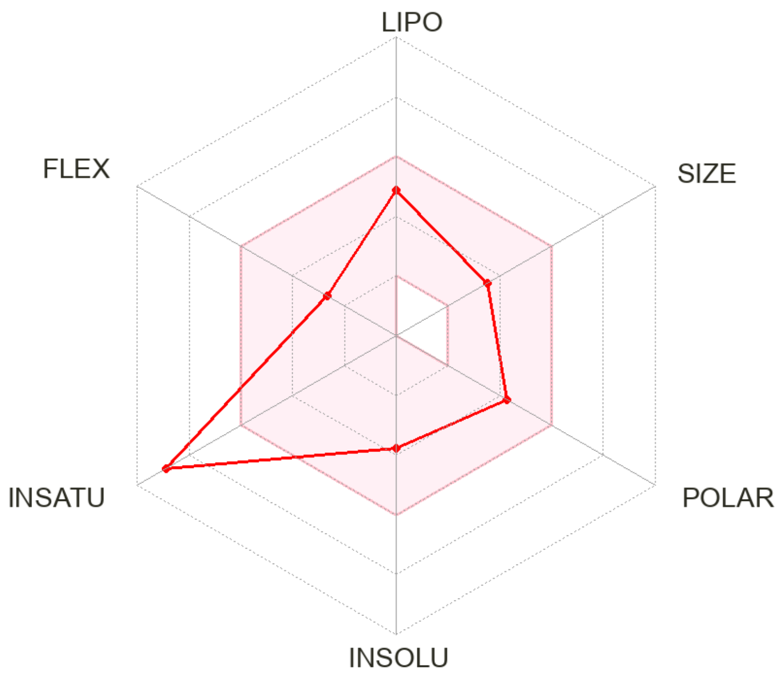

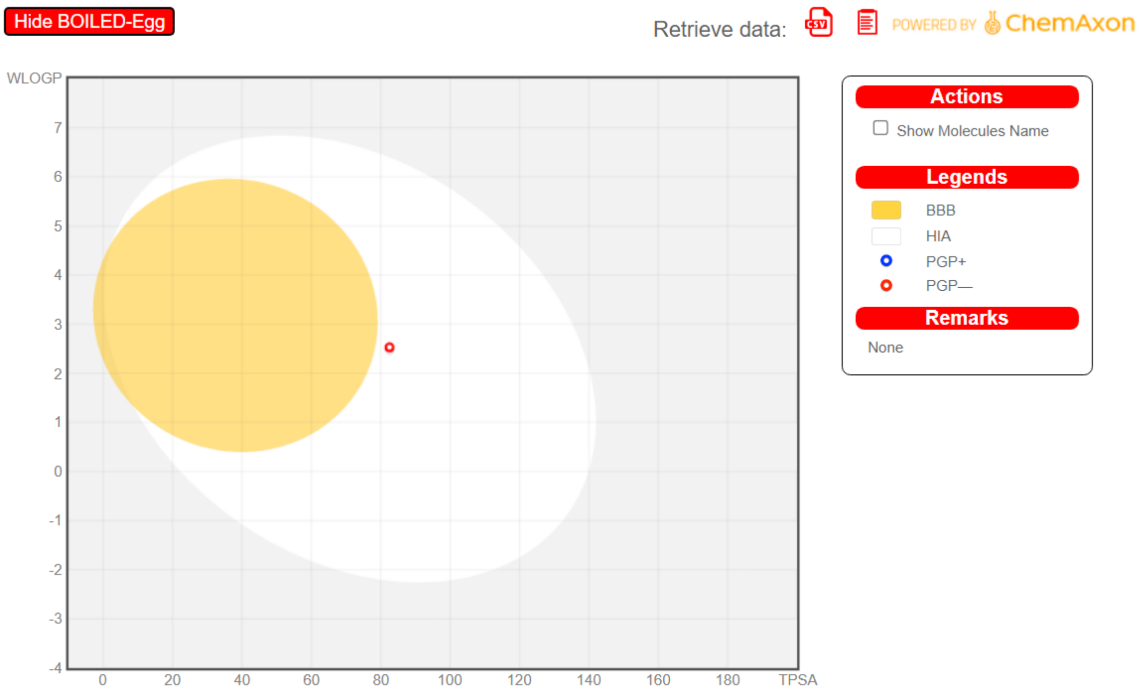

Figurw S12. Bioavailability radar and BOILED-Egg model of compound **1l** predicted by SwissADME program

Table S2. Physico-chemical properties and drug-likeness of compound **1a** predicted by SwissADME program

Canonical SMILES	Oc1ccccc1C=NNc1nc2c([nH]1)cccc2
Formula	C14H12N4O
MW	252.27
#Heavy atoms	19
#Aromatic heavy atoms	15
Fraction Csp3	0
#Rotatable bonds	3
#H-bond acceptors	3
#H-bond donors	3
MR	75.59
TPSA	73.3
iLOGP	0.82
XLOGP3	3.08
WLOGP	2.52
MLOGP	1.92
Silicos-IT Log P	2.59
Consensus Log P	2.19
ESOL Log S	-3.73
ESOL Solubility (mg/ml)	4.69E-02
ESOL Solubility (mol/l)	1.86E-04
ESOL Class	Soluble
Ali Log S	-4.29
Ali Solubility (mg/ml)	1.30E-02
Ali Solubility (mol/l)	5.17E-05
Ali Class	Moderately soluble
Silicos-IT LogSw	-5
Silicos-IT Solubility (mg/ml)	2.51E-03
Silicos-IT Solubility (mol/l)	9.93E-06
Silicos-IT class	Moderately soluble
GI absorption	High
BBB permeant	Yes
Pgp substrate	No
CYP1A2 inhibitor	Yes
CYP2C19 inhibitor	No
CYP2C9 inhibitor	No
CYP2D6 inhibitor	No
CYP3A4 inhibitor	No
log Kp (cm/s)	-5.65
Lipinski #violations	0
Ghose #violations	0

Veber #violations	0
Egan #violations	0
Muegge #violations	0
Bioavailability Score	0.55
PAINS #alerts	1
Brenk #alerts	1
Leadlikeness #violations	0
Synthetic Accessibility	2.52

Table S3. Physico-chemical properties and drug-likeness of compound **1b** predicted by SwissADME program

Canonical SMILES	Oc1cccc(c1O)C=NNc1nc2c([nH]1)cccc2
Formula	C14H12N4O2
MW	268.27
#Heavy atoms	20
#Aromatic heavy atoms	15
Fraction Csp3	0
#Rotatable bonds	3
#H-bond acceptors	4
#H-bond donors	4
MR	77.62
TPSA	93.53
iLOGP	0.32
XLOGP3	2.73
WLOGP	2.23
MLOGP	1.38
Silicos-IT Log P	2.1
Consensus Log P	1.75
ESOL Log S	-3.58
ESOL Solubility (mg/ml)	7.05E-02
ESOL Solubility (mol/l)	2.63E-04
ESOL Class	Soluble
Ali Log S	-4.35
Ali Solubility (mg/ml)	1.20E-02
Ali Solubility (mol/l)	4.48E-05
Ali Class	Moderately soluble
Silicos-IT LogSw	-4.42
Silicos-IT Solubility (mg/ml)	1.01E-02
Silicos-IT Solubility (mol/l)	3.77E-05
Silicos-IT class	Moderately soluble
GI absorption	High

BBB permeant	No
Pgp substrate	No
CYP1A2 inhibitor	Yes
CYP2C19 inhibitor	No
CYP2C9 inhibitor	No
CYP2D6 inhibitor	No
CYP3A4 inhibitor	No
log Kp (cm/s)	-6
Lipinski #violations	0
Ghose #violations	0
Veber #violations	0
Egan #violations	0
Muegge #violations	0
Bioavailability Score	0.55
PAINS #alerts	2
Brenk #alerts	2
Leadlikeness #violations	0
Synthetic Accessibility	2.58

Table S4. Physico-chemical properties and drug-likeness of compound **1c** predicted by SwissADME program

Canonical SMILES	Oc1ccc(c(c1)O)C=NNc1nc2c([nH]1)cccc2
Formula	C14H12N4O2
MW	268.27
#Heavy atoms	20
#Aromatic heavy atoms	15
Fraction Csp3	0
#Rotatable bonds	3
#H-bond acceptors	4
#H-bond donors	4
MR	77.62
TPSA	93.53
iLOGP	0.51
XLOGP3	2.73
WLOGP	2.23
MLOGP	1.38
Silicos-IT Log P	2.1
Consensus Log P	1.79
ESOL Log S	-3.58
ESOL Solubility (mg/ml)	7.05E-02
ESOL Solubility (mol/l)	2.63E-04

ESOL Class	Soluble
Ali Log S	-4.35
Ali Solubility (mg/ml)	1.20E-02
Ali Solubility (mol/l)	4.48E-05
Ali Class	Moderately soluble
Silicos-IT LogSw	-4.42
Silicos-IT Solubility (mg/ml)	1.01E-02
Silicos-IT Solubility (mol/l)	3.77E-05
Silicos-IT class	Moderately soluble
GI absorption	High
BBB permeant	No
Pgp substrate	No
CYP1A2 inhibitor	Yes
CYP2C19 inhibitor	No
CYP2C9 inhibitor	No
CYP2D6 inhibitor	No
CYP3A4 inhibitor	No
log Kp (cm/s)	-6
Lipinski #violations	0
Ghose #violations	0
Veber #violations	0
Egan #violations	0
Muegge #violations	0
Bioavailability Score	0.55
PAINS #alerts	2
Brenk #alerts	1
Leadlikeness #violations	0
Synthetic Accessibility	2.58

Table S5. Physico-chemical properties and drug-likeness of compound **1d** predicted by SwissADME program

Canonical SMILES	Oc1ccc(cc1O)C=NNc1nc2c([nH]1)cccc2
Formula	C14H12N4O2
MW	268.27
#Heavy atoms	20
#Aromatic heavy atoms	15
Fraction Csp3	0
#Rotatable bonds	3
#H-bond acceptors	4
#H-bond donors	4
MR	77.62

TPSA	93.53
iLOGP	0.5
XLOGP3	2.73
WLOGP	2.23
MLOGP	1.38
Silicos-IT Log P	2.1
Consensus Log P	1.79
ESOL Log S	-3.58
ESOL Solubility (mg/ml)	7.05E-02
ESOL Solubility (mol/l)	2.63E-04
ESOL Class	Soluble
Ali Log S	-4.35
Ali Solubility (mg/ml)	1.20E-02
Ali Solubility (mol/l)	4.48E-05
Ali Class	Moderately soluble
Silicos-IT LogSw	-4.42
Silicos-IT Solubility (mg/ml)	1.01E-02
Silicos-IT Solubility (mol/l)	3.77E-05
Silicos-IT class	Moderately soluble
GI absorption	High
BBB permeant	No
Pgp substrate	No
CYP1A2 inhibitor	Yes
CYP2C19 inhibitor	No
CYP2C9 inhibitor	No
CYP2D6 inhibitor	No
CYP3A4 inhibitor	No
log Kp (cm/s)	-6
Lipinski #violations	0
Ghose #violations	0
Veber #violations	0
Egan #violations	0
Muegge #violations	0
Bioavailability Score	0.55
PAINS #alerts	2
Brenk #alerts	2
Leadlikeness #violations	0
Synthetic Accessibility	2.48

Table S6. Physico-chemical properties and drug-likeness of compound **1e** predicted by SwissADME program

Canonical SMILES	Oc1ccc(c(c1O)O)C=NNc1nc2c([nH]1)cccc2
Formula	C14H12N4O3
MW	284.27
#Heavy atoms	21
#Aromatic heavy atoms	15
Fraction Csp3	0
#Rotatable bonds	3
#H-bond acceptors	5
#H-bond donors	5
MR	79.64
TPSA	113.76
iLOGP	0.38
XLOGP3	2.37
WLOGP	1.93
MLOGP	0.85
Silicos-IT Log P	1.62
Consensus Log P	1.43
ESOL Log S	-3.43
ESOL Solubility (mg/ml)	1.07E-01
ESOL Solubility (mol/l)	3.75E-04
ESOL Class	Soluble
Ali Log S	-4.4
Ali Solubility (mg/ml)	1.13E-02
Ali Solubility (mol/l)	3.99E-05
Ali Class	Moderately soluble
Silicos-IT LogSw	-3.84
Silicos-IT Solubility (mg/ml)	4.07E-02
Silicos-IT Solubility (mol/l)	1.43E-04
Silicos-IT class	Soluble
GI absorption	High
BBB permeant	No
Pgp substrate	No
CYP1A2 inhibitor	Yes
CYP2C19 inhibitor	No
CYP2C9 inhibitor	No
CYP2D6 inhibitor	No
CYP3A4 inhibitor	No
log Kp (cm/s)	-6.35
Lipinski #violations	0
Ghose #violations	0
Veber #violations	0
Egan #violations	0
Muegge #violations	0

Bioavailability Score	0.55
PAINS #alerts	3
Brenk #alerts	2
Leadlikeness #violations	0
Synthetic Accessibility	2.64

Table S7. Physico-chemical properties and drug-likeness of compound **1f** predicted by SwissADME program

Canonical SMILES	COc1ccc(cc1)C=NNc1nc2c([nH]1)cccc2
Formula	C15H14N4O
MW	266.3
#Heavy atoms	20
#Aromatic heavy atoms	15
Fraction Csp3	0.07
#Rotatable bonds	4
#H-bond acceptors	3
#H-bond donors	2
MR	80.06
TPSA	62.3
iLOGP	1.47
XLOGP3	3.41
WLOGP	2.83
MLOGP	2.18
Silicos-IT Log P	3.1
Consensus Log P	2.6
ESOL Log S	-3.93
ESOL Solubility (mg/ml)	3.13E-02
ESOL Solubility (mol/l)	1.17E-04
ESOL Class	Soluble
Ali Log S	-4.4
Ali Solubility (mg/ml)	1.06E-02
Ali Solubility (mol/l)	4.00E-05
Ali Class	Moderately soluble
Silicos-IT LogSw	-5.7
Silicos-IT Solubility (mg/ml)	5.29E-04
Silicos-IT Solubility (mol/l)	1.99E-06
Silicos-IT class	Moderately soluble
GI absorption	High
BBB permeant	Yes
Pgp substrate	No
CYP1A2 inhibitor	Yes

CYP2C19 inhibitor	Yes
CYP2C9 inhibitor	No
CYP2D6 inhibitor	Yes
CYP3A4 inhibitor	No
log Kp (cm/s)	-5.5
Lipinski #violations	0
Ghose #violations	0
Veber #violations	0
Egan #violations	0
Muegge #violations	0
Bioavailability Score	0.55
PAINS #alerts	0
Brenk #alerts	1
Leadlikeness #violations	0
Synthetic Accessibility	2.47

Table S8. Physico-chemical properties and drug-likeness of compound **1g** predicted by SwissADME program

Canonical SMILES	COc1cccc(c1C=NNc1nc2c([nH]1)cccc2)OC
Formula	C16H16N4O2
MW	296.32
#Heavy atoms	22
#Aromatic heavy atoms	15
Fraction Csp3	0.12
#Rotatable bonds	5
#H-bond acceptors	4
#H-bond donors	2
MR	86.56
TPSA	71.53
iLOGP	1.59
XLOGP3	3.38
WLOGP	2.84
MLOGP	1.88
Silicos-IT Log P	3.14
Consensus Log P	2.57
ESOL Log S	-3.98
ESOL Solubility (mg/ml)	3.09E-02
ESOL Solubility (mol/l)	1.04E-04
ESOL Class	Soluble
Ali Log S	-4.56
Ali Solubility (mg/ml)	8.15E-03

Ali Solubility (mol/l)	2.75E-05
Ali Class	Moderately soluble
Silicos-IT LogSw	-5.82
Silicos-IT Solubility (mg/ml)	4.50E-04
Silicos-IT Solubility (mol/l)	1.52E-06
Silicos-IT class	Moderately soluble
GI absorption	High
BBB permeant	Yes
Pgp substrate	No
CYP1A2 inhibitor	Yes
CYP2C19 inhibitor	Yes
CYP2C9 inhibitor	No
CYP2D6 inhibitor	Yes
CYP3A4 inhibitor	No
log Kp (cm/s)	-5.71
Lipinski #violations	0
Ghose #violations	0
Veber #violations	0
Egan #violations	0
Muegge #violations	0
Bioavailability Score	0.55
PAINS #alerts	0
Brenk #alerts	1
Leadlikeness #violations	0
Synthetic Accessibility	2.79

Table S9. Physico-chemical properties and drug-likeness of compound **1h** predicted by SwissADME program

Canonical SMILES	COc1cc(C=NNc2nc3c([nH]2)cccc3)cc(c1)OC
Formula	C16H16N4O2
MW	296.32
#Heavy atoms	22
#Aromatic heavy atoms	15
Fraction Csp3	0.12
#Rotatable bonds	5
#H-bond acceptors	4
#H-bond donors	2
MR	86.56
TPSA	71.53
iLOGP	1.9
XLOGP3	3.15

WLOGP	2.84
MLOGP	1.88
Silicos-IT Log P	3.14
Consensus Log P	2.58
ESOL Log S	-3.84
ESOL Solubility (mg/ml)	4.32E-02
ESOL Solubility (mol/l)	1.46E-04
ESOL Class	Soluble
Ali Log S	-4.32
Ali Solubility (mg/ml)	1.41E-02
Ali Solubility (mol/l)	4.76E-05
Ali Class	Moderately soluble
Silicos-IT LogSw	-5.82
Silicos-IT Solubility (mg/ml)	4.50E-04
Silicos-IT Solubility (mol/l)	1.52E-06
Silicos-IT class	Moderately soluble
GI absorption	High
BBB permeant	Yes
Pgp substrate	No
CYP1A2 inhibitor	Yes
CYP2C19 inhibitor	Yes
CYP2C9 inhibitor	No
CYP2D6 inhibitor	Yes
CYP3A4 inhibitor	No
log Kp (cm/s)	-5.87
Lipinski #violations	0
Ghose #violations	0
Veber #violations	0
Egan #violations	0
Muegge #violations	0
Bioavailability Score	0.55
PAINS #alerts	0
Brenk #alerts	1
Leadlikeness #violations	0
Synthetic Accessibility	2.67

Table S10. Physico-chemical properties and drug-likeness of compound **1i** predicted by SwissADME program

Canonical SMILES	COc1cc(C=NNc2nc3c([nH]2)cccc3)cc(c1OC)OC
Formula	C17H18N4O3
MW	326.35

#Heavy atoms	24
#Aromatic heavy atoms	15
Fraction Csp3	0.18
#Rotatable bonds	6
#H-bond acceptors	5
#H-bond donors	2
MR	93.05
TPSA	80.76
iLOGP	1.87
XLOGP3	3.35
WLOGP	2.84
MLOGP	1.6
Silicos-IT Log P	3.19
Consensus Log P	2.57
ESOL Log S	-4.04
ESOL Solubility (mg/ml)	2.97E-02
ESOL Solubility (mol/l)	9.11E-05
ESOL Class	Moderately soluble
Ali Log S	-4.72
Ali Solubility (mg/ml)	6.17E-03
Ali Solubility (mol/l)	1.89E-05
Ali Class	Moderately soluble
Silicos-IT LogSw	-5.93
Silicos-IT Solubility (mg/ml)	3.82E-04
Silicos-IT Solubility (mol/l)	1.17E-06
Silicos-IT class	Moderately soluble
GI absorption	High
BBB permeant	No
Pgp substrate	No
CYP1A2 inhibitor	Yes
CYP2C19 inhibitor	No
CYP2C9 inhibitor	Yes
CYP2D6 inhibitor	Yes
CYP3A4 inhibitor	No
log Kp (cm/s)	-5.91
Lipinski #violations	0
Ghose #violations	0
Veber #violations	0
Egan #violations	0
Muegge #violations	0
Bioavailability Score	0.55
PAINS #alerts	0
Brenk #alerts	1

Leadlikeness #violations	0
Synthetic Accessibility	2.86

Table S11. Physico-chemical properties and drug-likeness of compound **1j** predicted by SwissADME program

Canonical SMILES	COc1cccc(c1O)C=NNc1nc2c([nH]1)cccc2
Formula	C15H14N4O2
MW	282.3
#Heavy atoms	21
#Aromatic heavy atoms	15
Fraction Csp3	0.07
#Rotatable bonds	4
#H-bond acceptors	4
#H-bond donors	3
MR	82.09
TPSA	82.53
iLOGP	1.39
XLOGP3	3.05
WLOGP	2.53
MLOGP	1.63
Silicos-IT Log P	2.62
Consensus Log P	2.25
ESOL Log S	-3.78
ESOL Solubility (mg/ml)	4.72E-02
ESOL Solubility (mol/l)	1.67E-04
ESOL Class	Soluble
Ali Log S	-4.45
Ali Solubility (mg/ml)	1.00E-02
Ali Solubility (mol/l)	3.55E-05
Ali Class	Moderately soluble
Silicos-IT LogSw	-5.12
Silicos-IT Solubility (mg/ml)	2.13E-03
Silicos-IT Solubility (mol/l)	7.55E-06
Silicos-IT class	Moderately soluble
GI absorption	High
BBB permeant	No
Pgp substrate	No
CYP1A2 inhibitor	Yes
CYP2C19 inhibitor	No
CYP2C9 inhibitor	No
CYP2D6 inhibitor	Yes

CYP3A4 inhibitor	No
log Kp (cm/s)	-5.86
Lipinski #violations	0
Ghose #violations	0
Veber #violations	0
Egan #violations	0
Muegge #violations	0
Bioavailability Score	0.55
PAINS #alerts	1
Brenk #alerts	1
Leadlikeness #violations	0
Synthetic Accessibility	2.65

Table S12. Physico-chemical properties and drug-likeness of compound **1k** predicted by SwissADME program

Canonical SMILES	COc1ccc(c(c1)O)C=NNc1nc2c([nH]1)cccc2
Formula	C15H14N4O2
MW	282.3
#Heavy atoms	21
#Aromatic heavy atoms	15
Fraction Csp3	0.07
#Rotatable bonds	4
#H-bond acceptors	4
#H-bond donors	3
MR	82.09
TPSA	82.53
iLOGP	1.28
XLOGP3	3.05
WLOGP	2.53
MLOGP	1.63
Silicos-IT Log P	2.62
Consensus Log P	2.22
ESOL Log S	-3.78
ESOL Solubility (mg/ml)	4.72E-02
ESOL Solubility (mol/l)	1.67E-04
ESOL Class	Soluble
Ali Log S	-4.45
Ali Solubility (mg/ml)	1.00E-02
Ali Solubility (mol/l)	3.55E-05
Ali Class	Moderately soluble
Silicos-IT LogSw	-5.12

Silicos-IT Solubility (mg/ml)	2.13E-03
Silicos-IT Solubility (mol/l)	7.55E-06
Silicos-IT class	Moderately soluble
GI absorption	High
BBB permeant	No
Pgp substrate	No
CYP1A2 inhibitor	Yes
CYP2C19 inhibitor	No
CYP2C9 inhibitor	No
CYP2D6 inhibitor	No
CYP3A4 inhibitor	No
log Kp (cm/s)	-5.86
Lipinski #violations	0
Ghose #violations	0
Veber #violations	0
Egan #violations	0
Muegge #violations	0
Bioavailability Score	0.55
PAINS #alerts	1
Brenk #alerts	1
Leadlikeness #violations	0
Synthetic Accessibility	2.66

Table S13. Physico-chemical properties and drug-likeness of compound **1l** predicted by SwissADME program

Canonical SMILES	COc1ccc(cc1O)C=NNc1nc2c([nH]1)cccc2
Formula	C15H14N4O2
MW	282.3
#Heavy atoms	21
#Aromatic heavy atoms	15
Fraction Csp3	0.07
#Rotatable bonds	4
#H-bond acceptors	4
#H-bond donors	3
MR	82.09
TPSA	82.53
iLOGP	2.18
XLOGP3	3.05
WLOGP	2.53
MLOGP	1.63
Silicos-IT Log P	2.62

Consensus Log P	2.4
ESOL Log S	-3.78
ESOL Solubility (mg/ml)	4.72E-02
ESOL Solubility (mol/l)	1.67E-04
ESOL Class	Soluble
Ali Log S	-4.45
Ali Solubility (mg/ml)	1.00E-02
Ali Solubility (mol/l)	3.55E-05
Ali Class	Moderately soluble
Silicos-IT LogSw	-5.12
Silicos-IT Solubility (mg/ml)	2.13E-03
Silicos-IT Solubility (mol/l)	7.55E-06
Silicos-IT class	Moderately soluble
GI absorption	High
BBB permeant	No
Pgp substrate	No
CYP1A2 inhibitor	Yes
CYP2C19 inhibitor	No
CYP2C9 inhibitor	No
CYP2D6 inhibitor	Yes
CYP3A4 inhibitor	No
log Kp (cm/s)	-5.86
Lipinski #violations	0
Ghose #violations	0
Veber #violations	0
Egan #violations	0
Muegge #violations	0
Bioavailability Score	0.55
PAINS #alerts	0
Brenk #alerts	1
Leadlikeness #violations	0
Synthetic Accessibility	2.55
